# Byproducts of Globe Artichoke and Cauliflower Production as a New Source of Bioactive Compounds in the Green Economy Perspective: An NMR Study

**DOI:** 10.3390/molecules28031363

**Published:** 2023-01-31

**Authors:** Cinzia Ingallina, Giacomo Di Matteo, Mattia Spano, Erica Acciaro, Enio Campiglia, Luisa Mannina, Anatoly Petrovich Sobolev

**Affiliations:** 1 Food Chemistry Lab, Department of Chemistry and Technology of Drugs, Sapienza University of Rome, P. le Aldo Moro 5, 00185 Rome, Italy; 2 “Annalaura Segre” Magnetic Resonance Laboratory, Institute for Biological Systems, CNR, Via Salaria, Km 29,300, 00015 Monterotondo, Italy; 3 Department of Agricultural and Forest Sciences, University of Tuscia, Via San Camillo de Lellis, snc, 01100 Viterbo, Italy

**Keywords:** globe artichoke, cauliflower, byproducts, NMR, sesquiterpene lactones, methiin, glucosinolates

## Abstract

The recovery of bioactive compounds from crop byproducts leads to a new perspective way of waste reutilization as a part of the circular economy. The present study aimed at an exhaustive metabolite profile characterization of globe artichoke and cauliflower byproducts (leaves, stalks, and florets for cauliflower only) as a prerequisite for their valorization and future implementations. The metabolite profile of aqueous and organic extracts of byproducts was analyzed using the NMR-based metabolomics approach. Free amino acids, organic acids, sugars, polyols, polyphenols, amines, glucosinolates, fatty acids, phospho- and galactolipids, sterols, and sesquiterpene lactones were identified and quantified. In particular, globe artichoke byproducts are a source of health-beneficial compounds including *chiro*-inositol (up to 10.1 mg/g), *scyllo*-inositol (up to 1.8 mg/g), sesquiterpene lactones (cynaropicrin, grosheimin, dehydrocynaropicrin, up to 45.5 mg/g in total), inulins, and chlorogenic acid (up to 7.5 mg/g), whereas cauliflower byproducts enclose bioactive sulfur-containing compounds *S*-methyl-L-cysteine *S*-oxide (methiin, up to 20.7 mg/g) and glucosinolates. A variable content of all metabolites was observed depending on the crop type (globe artichoke vs. cauliflower) and the plant part (leaves vs. stalks). The results here reported can be potentially used in different ways, including the formulation of new plant biostimulants and food supplements.

## 1. Introduction

Worldwide, thousands of tons of agricultural food waste and byproducts are generated along the fruit and vegetable supply chain, which is one of the categories with the highest wastage rate (approximately 45%) [1]. However, vegetable byproducts and waste still represent a valuable raw material as a source of compounds, both nutrients and secondary metabolites, that can be used for different purposes. Many efforts are currently being made for the valorization of byproducts and wastes from plant sources in contrast to conventional or traditional food waste management practices [2]. Novel and emerging valorization approaches are investigated with the aim to turn the waste into a high value-added resource, combining sustainability, high technology, and cost reduction, according to UN Agenda 2030 goals for Sustainable Development [3]. Indeed, the greater interest in agri-food waste mainly relies in the recovery of bioactive compounds, such as polyphenols, pigments, terpenes, anthocyanins, alkaloids for the discovery and development of new pharmaceuticals, nutraceuticals, and cosmetics ingredients [4,5]; dietary fiber, polysaccharides and polymers, such as pectin, for the development of new functional foods [6] and/or biomaterials, such as cellulose, lignin, [7,8] and, to a lesser extent, proteins for the production of bioactive peptides [9,10].

In this context, governments interested in the reutilization and valorization of local agri-food waste often support the development of innovations in this field. It is the case of the Italian Lazio Region government that funded the research project entitled “Valorization of agri-food waste from the fruit and vegetable sector of Lazio region: from biostimulants for agriculture to supplements for human health”. In the frame of this project, byproducts of two crops, globe artichoke and cauliflower, important to the local economy, were chosen. According to FAO statistical data [11], Italy’s annual production of artichoke and cauliflower is about 3.8 × 10^8^ kg and 3.6 × 10^8^ kg, respectively. Considering that the major part of raw material (approximately 80% in the case of globe artichoke [12] and up to 60% in the case of cauliflower [13]) ends up as solid waste or left in the field, it is clear that a huge amount of waste is continuously generated, giving rise to environmental risks. In the case of globe artichoke, the main byproducts (about 65% of all wastes, [12]) are leaves and stalks, whereas cauliflower byproducts include also one part of the florets. A number of researches were dedicated to the valorization of artichoke byproducts as a source of valuable bioactive compounds, that can be extracted and re-utilized (see [14,15] and reference therein). Until now, the chemical characterization of these byproducts has been focused on single compounds or just a number of components belonging to a specific chemical class. Long-chain inulins have been recovered from bracts and stalks [16], roots [12], and external bracts [17]; polyphenols from leaves [18], stalks, and roots [12], or from bracts [19] have been extracted and characterized. Moreover, inositol isomers from the residual biomass of artichoke have been isolated [17,20]. Extracts with high antioxidant activity can be obtained from artichoke industrial byproducts [21]. After the extraction of bioactive compounds, the residual biomass represents a feedstock for energy production, for example by anaerobic digestion [22]. With respect to globe artichoke, cauliflower byproducts valorization received substantially minor attention with only a few examples relative to the extraction of phenolic antioxidants [23,24,25], dietary fibers [26], and peptide hydrolysates [27].

The analysis of reported studies shows that the valorization of globe artichoke and cauliflower byproducts require an integrated approach not limited to the recovery of single compounds or components of a specific chemical class, but considers the byproduct matrix as a source of different classes of bioactive compounds to be recovered altogether. Here, the untargeted NMR approach is proposed for the valorization of globe artichoke and cauliflower byproducts in a comprehensive way. NMR spectroscopy [28] has been successively applied for the comprehensive metabolite composition analysis of different plant tissues [29] including leaves [30], shoots [31], flowers [32], roots [33], tubers [34], and fruits [35]. Moreover, NMR spectroscopy has been already applied for the investigation of metabolite profiles of eatable parts of globe artichoke (entire heads or heads divided in external bracts and heart tissues) [36,37,38] and cauliflower [39], whereas a comprehensive NMR-based metabolite profiling of non-eatable parts of globe artichoke and cauliflower is still absent. The present study aimed to fill this gap by extending NMR characterization to byproducts, including also the identification of apolar metabolite profiles disregarded in the previous NMR studies. An exhaustive NMR characterization of byproducts is an important starting point for their valorization and for future implementations in the formulation of new products, such as plant growth bio-stimulants and food supplements.

## 2. Results

Here, the results regarding globe artichoke and cauliflower byproducts are reported separately.

### 2.1. Globe Artichoke Byproducts Metabolite Profiles

#### 2.1.1. Water-Soluble Metabolites: Assignment of NMR Spectra and Metabolite Identification

The assignments of ^1^H and ^13^C NMR spectra of leaves and stalks aqueous extracts reported in Table 1 were based on mentioned studies [36,37,38] and were confirmed by 2D NMR experiments (^1^H-^1^H TOCSY, ^1^H-^13^C HSQC, ^1^H-^13^C HMBC, Figures S1–S7 in the Supplementary Materials) and by comparison with corresponding NMR data of pure standard compounds from BMRB database [40].

The differences in the metabolite profile of leaves and stalks, with respect to heads, were observed. For example, the signals of rhamnose [36], shikimic acid, and gallic acid previously identified in head extracts of globe artichoke [37], were absent in leaves and stalks, whereas additional signals have been observed corresponding to metabolites not previously described including amines (ethanolamine, glycerophosphorylcholine, phosphorylcholine), histidine, acetic acid and two isomers of *myo*-inositol (*scyllo*-inositol and *chiro*-inositol).

Ethanolamine was identified thanks to its characteristic triplet signal at 3.15 ppm from CH_2_ group, whereas two derivatives of choline, namely, phosphorylcholine and glycerophosphorylcholine, show characteristic singlet signals of N(CH_3_)_3_ group at 3.23 and 3.24 ppm, respectively. Two-dimensional ^1^H-^13^C HSQC spectrum was used to identify all other ^1^H and ^13^C signals from CH_2_ and CH groups of all these amines definitely confirming the assignment (Table 1, Figure S1).

The characteristic broad singlet at 8.13 ppm (^1^H) correlated in the ^1^H-^1^H TOCSY map with the signal at 7.18 ppm was assigned to CH-2 group of histidine heterocycle. The acetate was identified thanks to its ^1^H singlet signal at 1.93 ppm correlated with the corresponding ^13^C signal at 24.4 ppm due to CH_3_ group.

The ^1^H and ^13^C NMR signals of *scyllo*-inositol and *chiro*-inositol were assigned using the literature data [41,42]. Usually, the presence of *chiro*-inositol signals in ^1^H NMR spectra of plant extracts is hardly visible due to the strong overlapping with the signals from common sugars (glucose, fructose, and sucrose). In fact, the signal of CH-3,4 of *chiro*-inositol at 3.59 ppm is partially overlapped with that from CH-1 of β-fructofuranose at 3.60 ppm, the signal at 3.76 ppm of CH-2,5 group (*chiro*-inositol) is masked by the signals from CH-3 of sucrose (3.77 ppm), and CH-6 of α- and β-glucose (3.78 and 3.74 ppm, correspondingly), whereas the signal at 4.05 ppm of CH-1,6 is masked by CH-4′ of sucrose (4.06 ppm) and CH-6 of β-fructopyranose (4.03 ppm). In our case, the multiplet signal of CH-3,4 group of *chiro*-inositol at 3.59 ppm was chosen for the quantification because it was only partially overlapped with other signals (Figure S8) and the level of *chiro*-inositol in leaves and stalks was comparable with the level of sugars making feasible the direct quantification.

In the case of *scyllo*-inositol, its ^1^H NMR spectrum consists of a single singlet signal at 3.36 ppm usually not overlapped with the signals of other molecules except the methanol signal. The correctness of the assignment was verified by the chemical shift value of the corresponding ^13^C signal in ^1^H-^13^C HSQC map observed at 74.6 ppm in accordance with the literature [42]. This information confirms the presence of *scyllo*-inositol excluding the presence of methanol whose ^13^C NMR signal is at 49.3 ppm.

Among the different mono- and di-caffeoylquinic acid derivatives characteristic of globe artichoke [36], only 5-caffeoylquinic (chlorogenic) acid and 3-caffeoylquinic (neochlorogenic) acid were identified in the NMR spectra of leaves and stalk extracts. It is noteworthy that the nomenclature and atom numbering of the ring of quinic acid moiety in caffeoylquinic acids reported in literature are sometimes confusing and misleading. The NMR assignment of chlorogenic and neochlorogenic acid here reported (Table 1) leaned upon correct nomenclature and atom numbering reported by [43,44]. The present assignment is related to the most abundant components and does not exclude the presence of dicaffeoylquinic acids (such as cynarine) as lower-level components. Moreover, the presence of a minor fraction of flavonoids reported in the literature for heads extracts [36,37] cannot be excluded, since the related ^1^H NMR spectral region (6.4–7.6 ppm) is too overlapped to separate the corresponding signals.

#### 2.1.2. Organic Fraction: Sesquiterpene Lactones and Other Metabolites

**Figure 1:**
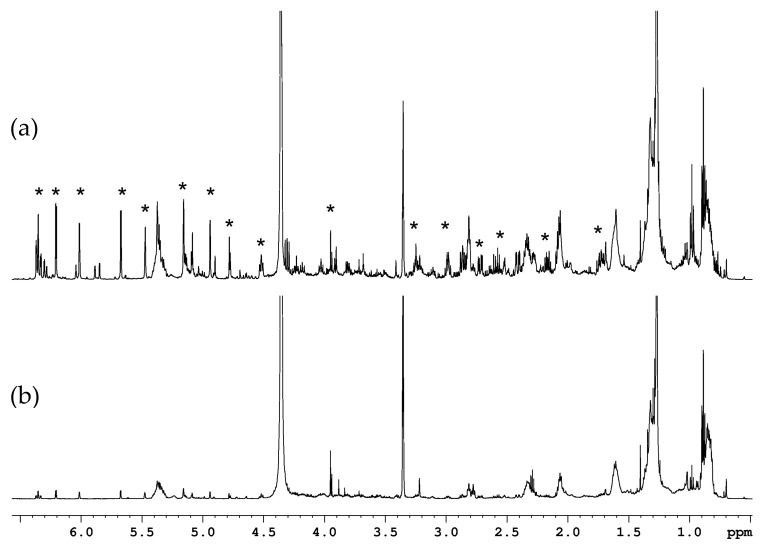
^1^H NMR spectra of globe artichoke leaves (**a**) and stalks (**b**) chloroform extracts. Solvent: CDCl_3_/CD_3_OD 2:1 *v*/*v*. Asterisks indicate cynaropicrin signals.

The ^1^H NMR spectrum of the chloroform fraction of leaf extracts is dominated by the signals of sesquiterpene lactones (STLs), (Figure 1), which are spread in the wide spectral range from 1.2 to 6.5 ppm. The characteristic ^1^H signals of CH_2_=C double bond fragments are observable in the 4.7–6.5 ppm range. The step-by-step assignment of the NMR signals with the identification of the corresponding three different STLs directly in the mixture was possible thanks to the analysis of 2D NMR experiments: ^1^H-^1^H correlations in TOCSY, ^1^H-^13^C direct and indirect correlations in HSQC and HMBC experiments (Figures S9–S14 in the Supplementary Materials).

**Figure 2:**
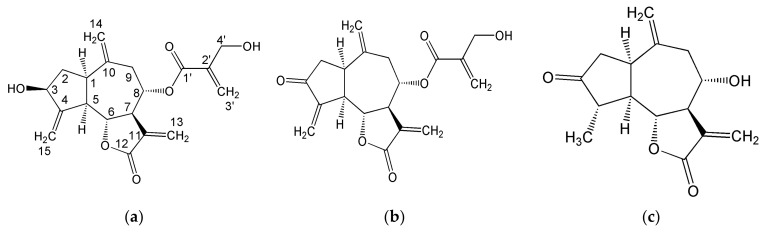

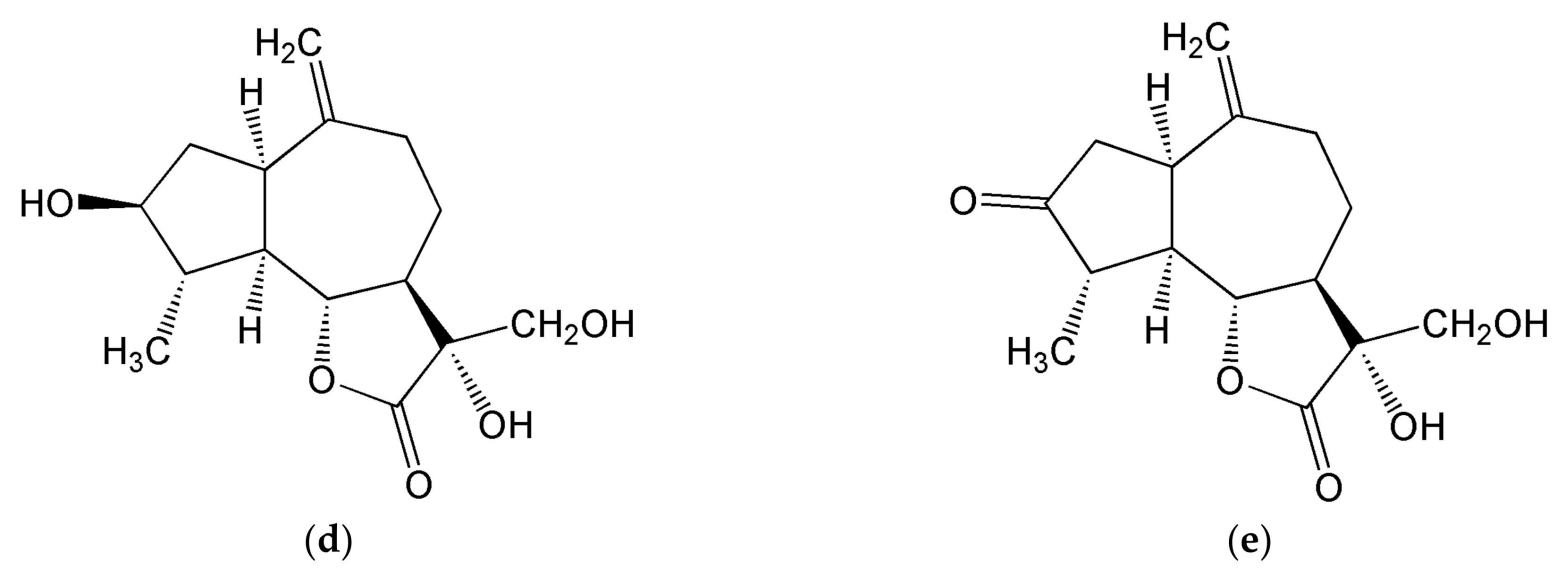
Structures of sesquiterpene lactones: (**a**) cynaropicrin; (**b**) dehydrocynaropicrin; (**c**) grosheimin; (**d**) cynaratriol; (**e**) 8-deoxy-11,13-dihydroxygrosheimin.

The STLs signals assignment partially rested on literature ^1^H and ^13^C NMR data (see below), obtained in similar (CDCl_3_ or deuterated methanol) but not identical solvents. In the present study a 2:1 *v*/*v* mixture of CDCl_3_ and CD_3_OD was used instead of a single solvent (CDCl_3_ or CD_3_OD). The mixture was chosen as one of the most suitable solvents to obtain narrow signals of lipidic components from vegetable extracts. In particular, cynaropicrin (Figure 2a, Table 2) was identified using NMR assignments in methanol [45] and chloroform [46] solutions. Dehydrocynaropicrin (Figure 2b, Table 2) [47] and grosheimin (Figure 2c, Table 2) [48,49] were identified by comparison our NMR data with the literature relative to chloroform solutions.

The key feature that characterizes both cynaropicrin and dehydrocynaropicrin is the presence of 2-(hydroxymethyl)acrylic acid moiety that forms an ester with C(8)-OH group. The corresponding ^1^H and ^13^C NMR signals were identified, see Table 2. The ^13^C NMR signals of C(3)=O ketone group in dehydrocynaropicrin and grosheimin at 205.2 and 220.8 ppm, respectively, were assigned thanks to long-range ^1^H-^13^C correlations in ^1^H-^13^C HMBC map.

Besides sesquiterpene lactones specific for globe artichoke, other metabolites such as fatty acids, sterols, pheophytins, phospho- and galactolipids, were also identified (Table 3). These compounds are typical components of lipidic fraction present in many vegetable tissues including leaves, stalks, fruits, and florets. The NMR assignment of these metabolites was based on our previous NMR studies of organic extracts from lettuce leaves [30], celery [50], and kiwifruits [51].

Fatty acid chains can be separated in NMR spectra according to the number of double bonds (mono-, di- and polyunsaturated fatty acids chains) but not according to the chain length; homologous fatty acids (like stearic vs. palmitic) cannot be distinguished. Table 3 reports the assignment of diunsaturated (linoleic type) and triunsaturated (linolenic type) fatty chains spectra. These fatty chains can be readily distinguished and quantified due to the presence of specific signals from bis-allylic methylene groups (CH_2_ groups between two *cis*-double bonds) at 2.78 and 2.81 ppm for linoleic and linolenic fatty chains, respectively. In the case of monounsaturated acids, the ^1^H NMR signals of their double bonds and allylic protons coincide with those from linoleic and linolenic acids, whereas other signals are overlapped with those from saturated fatty acids, therefore monounsaturated and saturated fatty chains were quantified together.

Three sterols, namely, β-sitosterol, campesterol, and stigmasterol, were also identified (Table 3). The characteristic singlet signal at 0.70 ppm is due to 18-CH_3_ group of both campesterol and β-sitosterol that cannot be separated in the ^1^H NMR spectra and were quantified as a sum.

The corresponding 18-CH_3_ signal of stigmasterol was observed separately at 0.72 ppm. The presence of a triterpenoid compound squalene, a precursor for synthesis of plant sterols, was evident due to the double bond CH signal at 5.12 ppm and other signals reported in Table 3.

Phosphatidylcholine and phosphatidylethnolamine were identified thanks to the signals of N(CH_3_)_3_ and CH_2_NH_2_ groups at 3.23 and 3.16 ppm, respectively. Unfortunately, the corresponding ^1^H spectral region was overlapped with the signals from other compounds, in particularly sesquiterpene lactones, hindering the quantification of phospholipids. The doublet at 4.91 ppm due to the anomeric proton of galactosyl ring indicated the presence of digalactolipids, typical for plant cells. Finally, chlorophylls from leaves during the extraction have lost magnesium ions and transformed into pheophytins a and b types readily observed in the spectra due to the characteristic proton signals in the range from 8 to 10 ppm from tetrapyrrole structure, Table 3.

#### 2.1.3. Metabolite Quantification

The quantification of the identified water-soluble metabolites was performed by the integration of corresponding selected ^1^H NMR signals. In a few cases, the strong overlapping of characteristic signals with the signals of other metabolites hampered the integration and quantification as in the case of arginine, GABA, lysine, tyrosine and neochlorogenic acid. The results of quantification for the aqueous extracts are reported in Table 4.

All amino acids, except alanine, were more abundant in leaves than in stalks. Asparagine was the most abundant amino acid in all artichoke byproducts.

Among organic acids, the highest level was shown by malic and quinic acids followed by citric acid in leaves and succinic acid in stalks. The content of malic and quinic acids in leaves and stalks was comparable, whereas the level of citric acid in leaves was about 10 times higher than in stalks.

The total sugar content (mostly glucose, fructose, and sucrose) in stalks was about seven times higher than in leaves; all sugars in stalks were 5–10 times more abundant than in leaves. The inulin level in stalks was approximately ten times higher than in leaves. Among three isomers of cyclic polyols, *chiro*-inositol was the most abundant in all byproducts followed by *myo*-inositol in stalks and *scyllo*-inositol in leaves.

Higher levels of choline and its derivatives were observed in leaves with respect to stalks; on the contrary, ethanolamine level was higher in stalks. Leaves were also characterized by a higher content of trigonelline, uridine and chlorogenic acid (5-caffeoylquinic acid). As mentioned previously, chlorogenic acid was the most abundant component among polyphenols, and other polyphenols were not quantified due to relatively low levels and strong overlapping of the corresponding signals.

The content of metabolites in the organic fraction of artichoke byproducts is reported in Table 5. Notably, leaves with respect to stalks showed a drastically higher level (about ten times) of sesquiterpene lactones, with cynaropicrin as the most abundant one (more than 60% of sesquiterpene lactones fraction) followed by grosheimin and dehydrocynaropicrin. Dehydrocynaropicrin was absent in stalks. The total content of sesquiterpene lactones in leaves was as high as 45.5 mg/g of DW (4.5% of dry weight). Leaves were also characterized by higher levels of sterols (β-sitosterol/campesterol and stigmasterol), squalene, digalactosyldiacylglycerol, and polyunsaturated fatty acids, especially triunsaturated linolenic fatty acid. Pheophytins a:b ratio (c.a. 3.7) reflects the chlorophyll a/b ratio in leaves and is typical for flowering plants. No pheophytin was present in the stalks extract.

### 2.2. Cauliflower Byproducts Metabolite Profile

**Figure 3:**
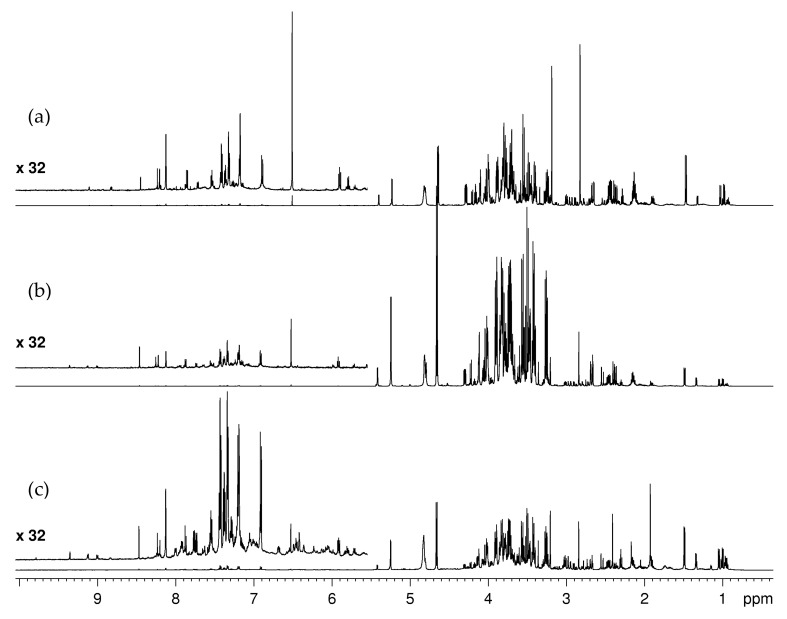
^1^H NMR spectra of cauliflower byproducts aqueous extracts: florets (**a**), stalks (**b**), leaves (**c**).

As in the case of globe artichoke byproducts, the NMR-based analysis of the metabolite composition of cauliflower byproducts is still absent in the literature. We included in the analysis not only leaves and stalks, but also florets that partially can end up in waste during the production cycle. Both aqueous and organic fractions of all three types of tissues were examined by NMR. The assignment of NMR spectra (Figure 3) was based on ^1^H and ^13^C NMR metabolite profiling of the eatable part (floret) reported in the literature [39] and the same set of 2D NMR experiments used for globe artichoke extracts (Figures S15–S23 in the Supplementary Materials).

#### 2.2.1. Assignment of *S*-Methyl-L-Cysteine-Sulfoxide (Methiin) in Aqueous Extracts

**Figure 4:**
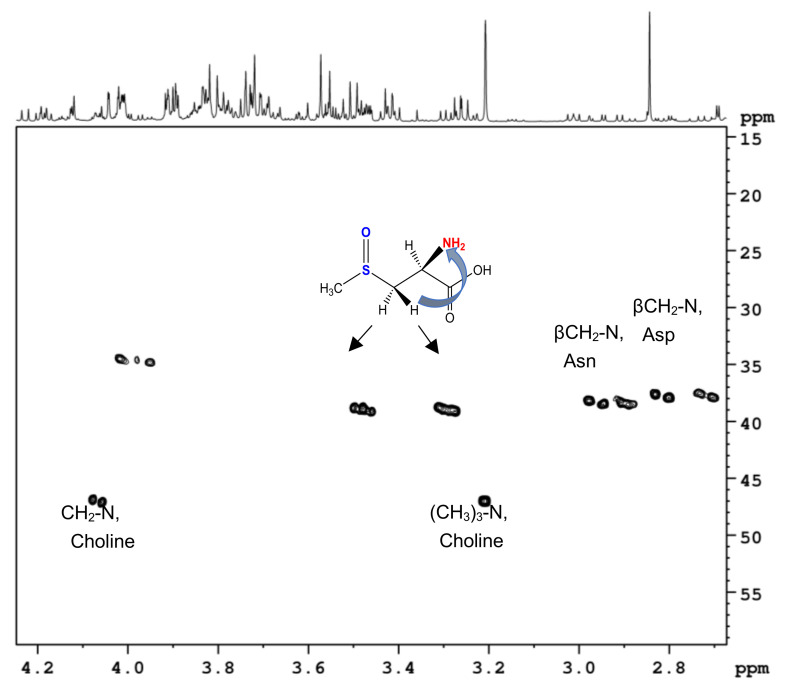
Selected region of ^1^H-^15^N HMBC NMR spectrum of cauliflower water-soluble metabolite fraction (florets extract).

All identified amino acids (Table 1) were already noticed in the literature [39], except one. Here, the presence of *S*-methyl-L-cysteine-sulfoxide (methiin) in the NMR spectra of aqueous extracts of cauliflower is reported for the first time. The identification of methiin had begun from the observation of the intense singlet signal at 2.84 ppm in the proton spectra of all types of cauliflower byproducts. According to ^1^H and ^13^C chemical shifts, this signal clearly belongs to an isolated methyl group CH_3_-X, where X could be a heteroatom (N or S). Heterocorrelation ^1^H-^13^C experiments showed the presence of CH_2_, CH and COOH groups in the molecule (Table 1); moreover, two protons of the methylene group were not chemically equivalent, indicating that the neighbor CH group was a chiral center. Therefore, the first hypothesis was the structure CH_3_-NH-CH_2_-CH(COOH)(NH_2_) known as β-*N*-methylaminoalanine. It is a non-protein amino acid produced by diverse cyanobacteria, dinoflagellates and diatoms [52] and a neurotoxin suspected to cause human neurodegenerative diseases. Fortunately, the addition of the corresponding standard compound into the extracts, followed by NMR analysis did not confirm the hypothesis. In fact, the methyl group of β-*N*-methylaminoalanine has a similar but not identical ^1^H chemical sift (2.79 ppm vs. 2.84 ppm) at the experimental conditions applied. Considering that the presence of nitrogen atoms was only supposed, additional experimental evidence was necessary. The direct observation of ^15^N NMR was not possible due to a low concentration of corresponding compounds, but the indirect long-range correlation experiment ^1^H-^15^N HMBC was successfully performed (Figure 4). In this experiment, no correlation between the proton signal at 2.84 ppm and any ^15^N signal was observed, indicating that the corresponding CH_3_ group was not directly bound to any nitrogen atom. Additionally, the correlation between two proton signals of the CH_2_ group (at 3.47 and 3.28 ppm, ^1^H) and a nitrogen atom of an amino group at 38.8 ppm (^15^N) confirmed that the molecule was an α-amino acid. Considering all the data, the following structure was finally deduced: CH_3_-S(O)-CH_2_-CH(COOH)(NH_2_), *S*-methyl-L-cysteine-sulfoxide (methiin). The experimental ^1^H and ^13^C chemical shifts were in good agreement with the literature data for one of the diastereoisomers, namely, (*R*,*S*)-*S*-methyl-cysteine sulfoxide [53], a natural compound present in many cruciferous vegetables [54]. Moreover, thanks to its characteristic ^1^H NMR signals, methiin was identified in ^1^H NMR spectra of urines as a marker of cruciferous vegetable consumption [55].

#### 2.2.2. Glucosinolates

Four glucosinolates, namely, glucoiberin, glucobrassicin, sinigrin, and glucoraphanin typical for cauliflower [56] were identified in leaves aqueous extract. Glucoiberin assignment was based on the presence of the characteristic ^1^H doublet signal (*J_H-H_* = 9.8 Hz) from anomeric proton of β-glucose ring at 5.07 ppm and a singlet signal at 2.74 ppm due to the methyl-sulfoxide group, see Table 1. Other glucoiberin signals reported in Table 1 were overlapped in the ^1^H spectrum with the signals from other metabolites and their assignment was based on 2D experiments, NMR data from the corresponding pure standard compound and the literature data [57]. Glucobrassicin has been identified thanks to its characteristic ^1^H signal of the CH-4″ group from indol moiety at 7.76 ppm (doublet, *J_H-H_* = 8.0 Hz), Table 1. Other indol proton signals were overlapped with those from tryptophane. The ^1^H signal from the anomeric proton of glucobrassicin glucose ring at 4.84 ppm is masked by an intense signal of residual water protons, but it was clearly visible in ^1^H-^13^C HSQC spectrum. The assignment was confirmed by the standard addition and was consistent with the literature [58]. The ^1^H NMR signals of sinigrin and glucoraphanin were much lower with respect to glucoiberin and glucobrassicin and their concentration was not enough to observe the corresponding cross-peaks in ^1^H-^13^C 2D correlation spectra. Only partial assignment of ^1^H spectrum of sinigrin and glucoraphanin was achievable using available TOCSY correlations, see Table 1. The assignment was confirmed by the addition of the corresponding standards.

#### 2.2.3. Other Metabolites in Aqueous Extracts

In the case of organic acids, in addition to acetic, formic, fumaric, malic, pyruvic, and succinic acids identified previously [39], citric, lactic, and quinic acids were identified (Table 1) in the ^1^H NMR spectra. Citric acid was present in all cauliflower byproducts, whereas lactic and quinic acids were detected only in leaves. Pyroglutamic acid was also identified in leaves and florets extracts.

The main sugars (glucose, fructose, and sucrose) present in florets [39] were also present in leaves and stalks, whereas no signals of galactose and xylose were observed (Table 1). Stalks and leaves ^1^H NMR spectrum has shown the presence of two doublet signals at 5.00 and 5.44 ppm characteristic for α-galactose and α-glucose anomeric protons of raffinose trisaccharide not previously reported for cauliflower [39].

Finally, besides choline, three additional amines (ethanolamine, glycerophosphorylcholine, phosphorylcholine) and uridine were identified for the first time in all cauliflower byproducts (Table 1).

#### 2.2.4. Liposoluble Metabolites

In the case of liposoluble metabolites, no specific secondary metabolites, such as sesquiterpene lactones in globe artichoke, were identified. Only the typical components of plant lipidic fraction such as fatty acids, sterols, pheophytins, phospho- and galactolipids were identified in NMR spectra of chloroform extracts (Table 3 and Figure S24) using the same 2D NMR experiments (Figures S25–S33 in the Supplementary Materials) and literature data as for globe artichoke byproducts. It is noteworthy that stigmasterol was not observed in cauliflower byproduct extracts.

#### 2.2.5. Metabolite Quantification in Cauliflower Byproducts

Comparing three different byproducts, the total amino acid content was in the order florets (F) > leaves (L) > stalks (S) (Table 4). Glutamine was the most abundant amino acid in all byproducts followed by methiin in S and F, and Arg in L. Glutamine and methiin levels in F were about four times higher than in L and S. Moreover, methiin content of L and S was very similar. Ala, Asn, Asp, GABA, Glu, Gln, and methiin showed the highest content in F, whereas the highest Arg, Leu, Lys, Phe, Thr, Trp, Tyr, and Val levels were observed in L. His and Ile levels were similar in L and F.

In the case of organic acids, again, F showed the highest total content. Malic acid was the most abundant in F and S, followed by citric acid, while all other acids were low. The relative levels of organic acids in L were quite different, with citric acid as the most abundant one, followed by malic, succinic, and acetic acids. Comparing the byproducts, S showed the highest absolute quantity of citric acid with respect to F and L.

The highest total content of sugars was observed for S, whereas the lowest one was in the case of L. Glucose was the most abundant in all byproducts, followed by fructose. Among all three byproducts the highest glucose, fructose, sucrose, and raffinose levels were found in S. Raffinose, a trisaccharide composed of galactose, fructose, and glucose found in different vegetables, was not present in F. The content of *myo*-inositol was comparable in L and S, whereas it was not possible to quantify it in F due to low level and strong overlapping with the signals of other metabolites. Maximum levels of all amines (ethanolamine, choline, and phosphorylcholine) and uridine were observed in F, with choline as the most abundant. S was characterized by the lowest levels of choline and ethanolamine. Glycerophosphorylcholine quantification was possible only in S, while in F and L extracts its NMR signals were covered by the signals of other metabolites. Glucosinolate signals were present only in the L extract. Out of four identified glucosinolates only two of them, glucoiberin and glucobrassicin, were quantified, whereas the levels of glucoraphanin and sinigrin were too low for the quantification.

Among the liposoluble metabolites reported in Table 5, sterols (β-sitosterol/campesterol), and all types of fatty acids (saturated, mono- and polyunsaturated) were more abundant in F than in S and L, while the highest levels of squalene and digalactosyldiacylglycerol were observed in leaves. As in the case of globe artichoke, only leaves of cauliflower contained pheophytins with the a:b ratio about 5.4.

## 3. Discussion

According to the literature, globe artichoke [12,16,18,59,60,61] and cauliflower byproducts [23,24,25,26,27] can be a valuable source of bioactive health-beneficial compounds, but the mentioned studies seem to give a fragmented view and usually are limited to a specific class of bioactive compounds or nutrients; none of them aimed to obtain a comprehensive picture of metabolite composition, including as many different classes of metabolites in the analysis as possible. Consequently, so far, only scattered data on the content of some metabolites obtained by analyzing different matrices and not the same sample have been available. The NMR-based metabolite profiling enables one to circumvent this problem thanks to the unbiased sensitivity of NMR analysis to all classes of organic compounds. Considering the results presented here, it was possible to monitor in the same sample contemporarily the presence of compounds belonging to completely different chemical classes such as free amino acids, organic acids, sugars, polyols, polyphenols, amines, glucosinolates, fatty acids, lipids, sterols, and sesquiterpene lactones.

The results of quantitative NMR analysis can be compared with the previous analytical studies conducted using other methods including HPLC [12,16,20,59], UHPLC/Q-TOF-MS [18], FT-IR [12], and capillary-electrophoretic methods [62], anyway bearing in mind differences in methodology (extraction solvent’s composition, type of extraction, analytical method), tissue type, genetic background, and cultivation practices.

### 3.1. Globe Artichoke Byproducts

#### 3.1.1. Inositols

Three isomeric cyclic polyalcohols of the inositol family (*myo*-inositol, *chiro*-inositol, and *scyllo*-inositol) are typical components of globe artichoke. Numerous studies attributed to them insulin-mimetic properties with improving insulin resistance [63], antihiperglycemic [64] and hepatoprotective activities [65]. The content of *chiro*- and *scyllo*-inositols in leaves and in stalks was in perfect agreement with the literature data [20]. *chiro*-Inositol in leaves and in stalks (about 10.0 and 5.0 mg/g DW, respectively), was the most abundant with respect to other isomers. Leaves were also richer in *scyllo*-inositol (1.8 vs. 0.6 mg/g DW in leaves and stalks, respectively). On the contrary, the lowest level of *myo*-inositol observed in leaves (0.27 mg/g) was too small with respect to the literature data (1.7 mg/g DW) [20].

#### 3.1.2. Sesquiterpene Lactones

The NMR analysis of leaves and stalks organic extracts has shown the presence of sesquiterpene lactones (STL) belonging to guaianolides, an important class of bioactive compounds with an intense bitter taste and a number of health beneficial properties [15]. In particular, cynaropicrin, the most abundant component of this class of compounds in globe artichoke, is known to manifest antihyperlipidemic, antimalarial, antispasmodic, antitrypanosomal, anti-photoaging, and antitumoral actions [66]. According to Rouphael et al. [18], apart from cynaropicrin, dehydrocynaropicrin, and grosheimin, the other two STLs (cynaratriol, Figure 2d, and 8-deoxy-11,13-dihydroxygrosheimin, Figure 2e) can be present in a comparable amount in artichoke leaves. Both 8-deoxy-11,13-dihydroxygrosheimin and cynaratriol are characterized by the absence of the C(8) hydroxyl group and the presence of two additional OH groups attached to C(11) and C(13) carbon atoms. The NMR signals of CH_2_(8) and CH_2_OH(13) groups were not observed, evidencing that neither 8-deoxy-11,13-dihydroxygrosheimin nor cynaratriol was present. The content of STLs in artichoke leaves is variable and strongly influenced by the cultivar [18]. The available literature data on the 19 most representative cultivars grown in Europe [18] can be considered as a reference giving the range of possible variations of STL’s content. To compare our data with the literature, where the STL’s levels were measured with respect to fresh weight, our data were recalculated considering that the average water content in leaves was about 89%. The contents of cynaropicrin (247.5 mg/100 g FW) and grosheimin (97.2 mg/100 g FW) were in the reported range (4.5–800) and (6.8–1600), respectively. On the contrary, dehydrocynaropicrin level (65.0 mg/100 g FW) was higher than the upper limit (3.3–25.6) reported. No data on the content of STLs in the stalks of globe artichoke are available in the literature.

#### 3.1.3. Caffeoylquinic Acids

Among caffeoylquinic acids, chlorogenic acid was the most abundant. The content of chlorogenic acid in leaves (7.5 mg/g DW) was relatively high considering the literature data for different cultivars of globe artichoke, such as Spinoso di Palermo (0.7–2.1 mg/g DW) (Violetto di Sicilia (0.1–1.9 mg/g DW) [59], Blanca di Tudela (2.4 mg/g DW) [20], Madrigal (3.9 mg/g DW) [12]. In the case of stalks, chlorogenic acid level (2.3 mg/g DW) was comparable to that reported in the literature for the same cultivars: Spinoso di Palermo (0.5–4.8 mg/g DW) [59], Violetto di Sicilia (0.5–4.7 mg/g DW) [59], Blanca di Tudela (1.6 mg/g DW) [20], Madrigal (2.8 mg/g DW) [12]. This comparison with the literature data does not take into account the differences in the extraction conditions that can substantially influence the yield [67].

#### 3.1.4. Inulins

Inulins, known also as oligofructans, are dietary fibers converted by colon bacteria into short-chain fatty acids necessary to nourish colon cells and stimulate the immune system [68]. In our case, the quantification of inulins extracted in the solution was based on the integration of the characteristic ^1^H signal at 5.44 ppm from the anomeric proton of the glucose terminal ring. It was not possible to measure the mean degree of oligomerization and therefore the number of fructose units in the inulin chain. The molecular weight of 1-kestose, (containing just one glucose and two fructose units) was used for the calculation of inulin weight in the samples, consequently, the results obtained (0.44 and 4.14 mg/g DW in stalks and leaves, respectively), represented the lowest limit and the real weight could be higher. In the case of globe artichokes, leaves and stalks contain a markedly lower amount of inulin with respect to heads and roots, anyway its reported content can be as high as 37 mg/g DW in leaves and 29 mg/g DW in stalks [12]. In comparison with the literature, our values were too small indicating that the extraction protocol was probably not optimized for inulin extraction. In fact, it is known that inulin’s extraction yield is strikingly dependent on the temperature [12], and Bligh-Dyer extraction temperature (4 °C) was too low with respect to the optimal temperature range (60–85 °C) for inulin.

#### 3.1.5. Other Metabolites

Apart from the content of specific metabolites discussed above, the usual content of all other metabolites identified in the present work in globe artichoke byproducts (such as amino acids, organic acids, amines, fatty acids, and lipids), with some exceptions, has not been reported yet in the literature. The exceptions are related to quinic acid and common sugars (glucose, fructose, and sucrose) already quantified in the leaves and stalks of a few samples [20,67]. In particular, the content of quinic acid in Blanca di Tudela cultivar byproducts was lower than in our case (1.3 vs. 7.6 mg/g DW and 2.6 vs. 6.5 mg/g DW in stalks and leaves, respectively) [20]. The content of fructose, glucose, and sucrose in Blanca di Tudela cultivar byproducts [20] was comparable with those obtained in the present study. In both cases, the total sugar content in stalks was 5–7 times higher than in leaves, with glucose as the most abundant sugar in all byproducts.

### 3.2. Cauliflower ByProducts

As mentioned in the introduction, cauliflower byproducts valorization topic received substantially minor attention with respect to globe artichoke. Only the content of a few particular classes of metabolites, such as polyphenols [23,24], has been determined so far. As to the other classes of metabolites (amino acids, organic acids, sugars, and some lipids), their content in leaves and stalks is reported here for the first time. Considering the absence of literature data on cauliflower byproducts’ metabolite composition, our NMR data relative to florets, leaves, and stalks can be compared only with those of the eatable part of cauliflower (florets) measured by NMR previously [39]. In the case of amino acids, the levels of aspartic acid, isoleucine and threonine were comparable with those in the previous study, whereas the observed levels of other amino acids were either too small (arginine, lysine, glutamic acid, phenylalanine) or too high (glutamine, histidine, alanine, valine) with respect to the eatable part of cauliflower. In particular, the level of glutamine, the most abundant amino acid in all byproducts, was about five to two times higher; on the contrary, those of arginine and lysine were seven to two times lower with respect to the edible part.

It is noteworthy that the level of methiin, the second most abundant amino acid (not proteinogenic) after glutamine, was not reported in the above mentioned NMR study [39], probably because it was not identified. Methiin is a major *S*-alkyl-L-cysteine *S*-oxide found in *Brassicaceae* (such as broccoli, Brussels sprouts, cabbage, and cauliflower) and in the domesticated *Allioideae* with concentrations up to 1–2% of dry weight [54,62]. Health-beneficial properties of methiin were observed in animal model studies including its effects on hyperlipidemia, as an antidiabetic, and as an antimicrobial agent [69,70]. The highest methiin level (about 20.7 mg/g DW that corresponds to 2.0 mg/g FW) observed in florets was in good agreement with the literature data for the eatable part of cauliflower (2.8 mg/g FW) [62].

Besides methiin, cauliflower byproducts also include other sulfur-containing secondary metabolites, members of the glucosinolates family. Glucosinolates are present in all *Brassicaceae* plants and have a common basic structure consisting of a β-D-thioglucoside group, a sulfonated moiety, and a variable side chain derived from one of eight natural amino acids ([71] and references therein). Glucosinolates are precursors of other biologically active compounds (such as isothiocyanates) with a number of health-beneficial properties that include chemopreventive activity against cancer, the risk reduction of cardiovascular disease, neurodegeneration, diabetes, and other inflammatory disorders ([71] and references therein). As in the case of other metabolites, no literature data are yet available on the glucosinolates content in cauliflower byproducts. According to the literature, the edible part of cauliflower (florets) contains 16–151 mg of glucosinolates in 100 g of fresh tissue [71], but in our case, no glucosinolates were detected by NMR in florets and in stalks. Considering that cauliflower byproducts were supplied by a local farm, partial enzymatic hydrolysis of glucosinolates could take place in the span of time between the harvest and delivery of byproducts. Regardless, leaves have shown a good level of glucosinolates (8.13 μmol/g DW or 44.7 mg/100g FW) with glucoiberin as the most abundant (2.09 mg/g DW or 4.94 μmol/g DW), followed by glucobrassicin (1.43 mg/g DW or 3.2 μmol/g DW), Table 4. These levels of glucosinolates seem to be even higher than those reported in a recent work for the eatable part of cauliflower (0.5–1.8 μmol/g DW for glucoiberin, and 0.2 µmol/g DW for glucobrassicin) [56].

The metabolite profiling of both cauliflower and globe artichoke byproducts using the same NMR methodology gives us the opportunity to compare directly the metabolite composition of different tissues of two different plant crops. The results of this comparison indicate:

(1) Close similarity of qualitative composition of the most abundant metabolites (including sugars, amino acids, organic acids, amines, etc.), in all samples studied. For example, 17 out of 19 identified amino acids were observed in both crops’ byproducts (Table 1). Among sugars, only minor components (such as inulin in artichoke and raffinose in cauliflower) were crop-specific;
(2) The presence of crop-specific secondary metabolites. In the case of globe artichoke byproducts, the crop-specific metabolites included sesquiterpene lactones, stigmasterol, inositol isomers (*chiro*- and *scyllo*-inositols), inulin, chlorogenic and neochlorogenic acids, and glycine betaine, whereas only cauliflower’s byproducts contained methiin, glycine, glucosinolates, pyruvic acid, and raffinose;
(3) The drastic variation of metabolite levels occurs in crop- and tissue-specific manner. For example, dehydrocynaropicrin present in globe artichoke leaves was absent in stalks, whereas glucosinolates were found only in cauliflower leaves. For both crops, leaves were generally richer in amino acids and amines and poorer in sugars with respect to stalks. Florets (in the case of cauliflower) were even richer in amino acids and amines than leaves;
(4) The detailed analysis of metabolite profiling of a given byproduct can indicate the best strategies for the recovery and the reutilization of its constituents. Both water-soluble and apolar metabolite fractions contain bioactive compounds that can be extracted and reutilized in food supplements or as components of functional food. In the case of globe artichoke, there is a remarkable potential for new product development from byproducts as sources of inositol isomers, caffeoylquinic acids and inulin in water-soluble fraction and sesquiterpene lactones in organic fraction. Cauliflower byproducts can be utilized for the extraction of bioactive sulfur-containing compounds including *S*-methyl-L-cysteine *S*-oxide and glucosinolates.

## 4. Materials and Methods

### 4.1. Plant Material

White cauliflower byproducts (*Brassica oleracea*), mainly consist of leaves and, in less amount, stalks, florets and curd (editable part), representing approximately the 60% [13], were supplied by F.lli Calevi Alberto e Stefano (Viterbo, Lazio, Italy). Cauliflower plants were harvested during the period of head maturity (100–90 days after sowing).

Globe artichoke byproducts (*Cynara scolymus*) mainly consisted of leaves and stalks (approximately 80%) [11] were supplied by Azienda Agricola Sperlonga-SANVIDA (Sperlonga, Lazio, Italy). The byproducts of two years old globe artichoke plants were harvested.

After collection, fresh cauliflower and globe artichoke byproducts were lyophilized (−55 °C, 0.200 mbar) and ground in power. The dry tissue powder was stored at −80 °C before performing extraction procedures. The mean water content (in % by weight) in globe artichoke was 89% for leaves and 92.5% for stalks, whereas in the case of cauliflower the following values of water content were calculated: 87.3% for leaves, 91% for stalks, and 90% for florets.

### 4.2. Chemicals

Water (HPLC-grade) and β-*N*-methylaminoalanine were purchased from Sigma-Aldrich (Milan, Italy). Methanol (HPLC-grade), chloroform, K_2_HPO_3_, and KH_2_PO_3_ were obtained from Carlo Erba Reagenti (Milan, Italy). Deuterated solvents (D_2_O, CD_3_OD, CDCl_3_) and 3-(trimethylsilyl)-propionic-2,2,3,3-d4 acid sodium salt (TSP), were purchased from Eurisotop (Saint-Aubin, France).

### 4.3. Extraction Procedures

Bligh–Dyer extraction method [72] was applied to the lyophilized samples. In detail, about 100 mg of grinded lyophilized sample (leaves, stalks, or florets) was mixed sequentially with 3 mL of methanol/chloroform (2:1 *v*/*v*) mixture, followed by 0.8 mL distilled water, carefully shaking after each addition. The obtained monophasic system was sonicated for 10 min at room temperature. Then, the extract was sequentially added with 1 mL of chloroform and 1 mL of distilled water to obtain a biphasic system. The extract was then centrifuged (4200 rpm for 15 min at 25 °C) and the upper (hydroalcoholic) and lower (chloroform) phases were carefully separated. The extraction procedure was repeated two more times on the pellet to guarantee the complete extraction of compounds, afterwards, the separated fractions were pooled. Both hydroalcoholic and chloroform fractions were dried under a gentle N_2_ flow at room temperature until the solvent was completely evaporated. The dried phases were stored at −20 °C until further analyses. For every byproduct type *n* = 3 replicates were extracted and analyzed by NMR spectroscopy.

### 4.4. NMR Analysis

#### 4.4.1. NMR Samples Preparation

The dried hydroalcoholic fraction was dissolved in 0.75 mL of buffered D_2_O, (400 mM phosphate buffer, pH = 7.0) containing the internal standard for chemical shift referencing and quantification (2 mM TSP). The solution was clarified by centrifugation (4200 rpm for 1 min at room temperature) and 0.7 mL of supernatant was put in a standard 5 mm NMR tube.

The dried chloroform fraction was dissolved in 0.75 mL of CDCl_3_/CD_3_OD (2:1 *v*/*v*) mixture containing tetramethylsilane (TMS) as an internal standard and then transferred in a 5 mm NMR tube that was flame sealed.

#### 4.4.2. NMR Experiments

The NMR spectra of all extracts were recorded at 27 °C on a Bruker AVANCE III HD 600 spectrometer (Rheinstetten, Germany) operating at the proton frequency of 600.13 MHz and equipped with a Bruker multinuclear z-gradient inverse probehead.

Proton spectra were referenced to TSP signal (δ = 0.00 ppm) or TMS signal (δ = 0.00 ppm) for hydroalcoholic and chloroform fractions, respectively.

The ^1^H spectra of the hydroalcoholic fraction were acquired by co-adding 256 transients with a recycle delay of 7 s and using a 90° pulse of 12–15 μs, 32k data points and 10.2 ppm spectral window width. The residual HDO signal was suppressed using a soft pulse presaturation scheme (Bruker pulse program zgpr) during the relaxation delay. The ^1^H spectra of the chloroform fraction were acquired by co-adding 256 transients with a recycle delay of 5 s and using a 90° pulse of 10–11 μs, 32k data points and 13 ppm spectral window width. All proton spectra after acquisition were zero-filled to 64k data points, and Fourier transformed using 0.3 Hz exponential multiplication factor. Manual phase and baseline correction were performed.

For the quantification of metabolites, the selected signals listed in Table 1 and Table 3 were integrated and the integrals were normalized with respect to the integral of the internal standard signal at 0.0 ppm (TSP or TMS for aqueous or organic extracts, respectively).

2D NMR spectra of all extracts were acquired under the experimental conditions previously reported [73]. ^1^H–^1^H TOCSY experiments were carried out with a mixing time of 80 ms, ^1^H–^13^C HSQC experiments with a coupling constant ^1^*J*_(C-H)_ of 150 Hz, and ^1^H–^13^C HMBC experiments with a delay of 100 ms for the evolution of long-range couplings. ^1^H–^15^N HMBC experiment was acquired using 5805 and 12,163 Hz spectral width for ^1^H and ^15^N, respectively, with a delay of 125 ms for the evolution of long-range couplings. Chemical shifts were calibrated using TSP and indirect referencing of ^15^N [74].

## 5. Conclusions

The present study introduces the NMR methodological approach to a comprehensive analysis of metabolites present in globe artichoke and cauliflower byproducts, aimed at their valorization as a source of different classes of valuable health-beneficial bioactive compounds that still can be recovered and reutilized. The results of qualitative and quantitative NMR analyses are consistent with the available literature data on similar byproducts analyzed by other analytical methods (HPLC [12,16,20,59], UHPLC/Q-TOF-MS [18], FT-IR [12], and electrokinetic capillary chromatography [62]), indicating that NMR analysis is a valid alternative for the characterization of similar agri-food byproducts and waste. The developed NMR-based approach is an important starting point for the valorization of byproducts and future implementation of recovered bioactive compounds in the formulation of new products, such as plant growth bio-stimulants and food supplements.

## Figures and Tables

**Table 1:** Metabolites identified in aqueous extracts of cauliflower and globe artichoke byproducts. Relative assignments of ^1^H and ^13^C NMR signals are reported. L = leaves; S = stalks; F = florets; d = doublet; dd = doublet of doublets; m = multiplet; qd = quartet of doublets; s = singlet; t = triplet.

Metabolite	Assignment	δ ^1^H (ppm)	Multiplicity (*J*_H-H_, Hz)	δ ^13^C (ppm)	Globe Artichoke	Cauliflower
	Amino acids					
Alanine (Ala)	β-CH_3_	1.49 *	d (7.2)	17.2	L, S	L, S, F
	α-CH	3.80		51.5		
Arginine (Arg)	α-CH	3.78		55.1	L	L, S, F
	β-CH_2_	1.93		28.6		
	γ, γ′-CH_2_	1.74; 1.67 *		24.9		
	δ-CH_2_	3.24		41.5		
Asparagine (Asn)	β-CH	2.89	dd (16.9; 7.2)	35.6	L, S	L, S, F
	β′-CH	2.96 *	dd (16.9; 4.5)	35.6		
	α-CH	4.01		52.2		
Aspartate (Asp)	β-CH	2.71	dd (17.4; 8.1)	37.6	L, S	L, S, F
	β′-CH	2.80 *	dd (17.4; 3.8)	37.6		
	α-CH	3.91		53.2		
γ-Aminobutyric acid (GABA)	β-CH_2_	1.91		24.7	L, S	L, S, F
	α-CH_2_	2.30 *	t (7.4)	35.4		
	γ-CH_2_	3.02	t (7.6)	40.2		
Glycine (Gly)	α-CH_2_	3.57	s	42.5		L, F
Glutamate (Glu)	β, β′-CH_2_	2.13; 2.08	m	28.0	L, S	L, S, F
	γ-CH_2_	2.35 *	m	34.4		
	α-CH	3.77		55.5		
Glutamine (Gln)	β-CH_2_	2.15	m	27.3	L, S	L, S, F
	γ-CH_2_	2.46 *	m	31.8		
	α-CH	3.78		55.2		
Histidine (His)	β, β′-CH_2_	3.30; 3.24		28.2	L	L, S, F
	α-CH	4.02		55.4		
	CH-5	7.18		118.3		
	CH-2	8.13 *				
Isoleucine (Ile)	δ-CH_3_	0.94	t (7.4)	12.1	L, S	L, S, F
	γ-CH_3_	1.01 *	d (7.0)	15.7		
	γ, γ′-CH_2_	1.48; 1.27		25.5		
	β-CH	1.98		36.8		
	α-CH	3.68		60.5		
Leucine (Leu)	δ-CH_3_	0.96 *	d (6.2)	22.1	L, S	L, S, F
	δ′-CH_3_	0.97 *	d (6.0)	23.0		
	β-CH_2_	1.73		40.9		
	α-CH	3.74		54.4		
	γ-CH	1.70		25.2		
Lysine (Lys)	α-CH	3.77		55.3	L	L, F
	β-CH_2_	1.92		30.9		
	γ-CH_2_	1.49		22.5		
	δ-CH_2_	1.74		27.4		
	ε-CH_2_	3.04 *	t (7.6)	40.0		
*S*-Methyl-L-cysteine-*S*-oxide (Methiin)	α-CH	4.18	t (6.8)	51.8		L, S, F
	β-CH_2_	3.47; 3.28	dd (14.0; 7.2)	54.5		
	γ-CH_3_	2.84 *	s	39.1		
	COOH			173.1		
Phenylalanine (Phe)	β, β′-CH_2_	3.27; 3.16		37.4	L, S	L, S, F
	α-CH	4.00		56.9		
	CH-2,6	7.34	d (7.3)	130.5		
	CH-4	7.38	t (7.0)	128.7		
	CH-3,5	7.43 *	t (7.3)	130.2		
Pyroglutamic acid	β, β’-CH_2_	2.04; 2.51		26.3	L	L, F
	γ-CH_2_	2.40		30.7		
	α-CH	4.18		59.3		
Threonine (Thr)	γ-CH_3_	1.34 *	d (6.6)	20.5	L, S	L, S, F
	α-CH	3.61		61.4		
	β-CH	4.26	qd (6.6; 4.9)	66.9		
Tryptophan (Trp)	CH-4	7.73 *	d (7.9)	119.5	L	L, S, F
	CH-7	7.55	d (7.4)	113.0		
	CH-6	7.29	t (7.4)	123.2		
	CH-5	7.20		120.4		
	CH-2	7.34	s	126.2		
	α-CH	4.06		55.9		
	β, β′-CH_2_	3.48; 3.32		27.4		
Tyrosine (Tyr)	CH-2,6	7.20	d (8.5)	131.7	L	L, S, F
	CH-3,5	6.91 *	d (8.5)	116.9		
	α-CH	3.95		57.1		
	β, β′-CH_2_	3.19; 3.07		36.5		
Valine (Val)	γ-CH_3_	1.00	d (7.0)	17.7	L, S	L, S, F
	γ′-CH_3_	1.05 *	d (7.0)	19.0		
	β-CH	2.28		30.1		
	α-CH	3.62		61.3		
	Organic acids					
Acetic acid (AA)	αCH_3_	1.93 *	s	24.4	L, S	L, S, F
Citric acid (CA)	α, γ-CH	2.54 *; 2.68	d (15.5)	46.5	L, S	L, S, F
Formic acid (FA)	HCOO-	8.47 *	s	173.8	L, S	L, S, F
Fumaric acid (FumA)	α, β-HC=CH	6.53 *	s	136.5	L, S	L, S, F
Lactic acid (LA)	β-CH_3_	1.33 *	d (6.9)	21.1	S	L
	α-CH	4.12		69.6		
Malic acid (MA)	β-CH	2.68	dd (15.4; 3.2)	43.6	L, S	L, S, F
	β′-CH	2.39	dd (15.4; 10.0)	43.6		
	α-CH	4.30 *	dd (10.0; 3.2)	71.4		
Pyruvic acid (PA)	CH_3_	2.36	s	30.0		L, F
Quinic acid (QA)	C(OH)COOH			78.1	L, S	L
	2,2′-CH_2_	2.04; 1.97		38.5		
	6,6′-CH_2_	2.08; 1.88 *		41.8		
	CH-3	4.16		71.5		
	CH-4	3.56		76.3		
	CH-5	4.03		68.0		
Succinic acid (SA)	α, β-CH_2_	2.41 *	s	35.1	L, S	L, S, F
	Carbohydrates, polyols					
α-Glucose (α-Glc)	CH-1	5.25 *	d (3.8)	93.1	L, S	L, S, F
	CH-2	3.55		72.4		
	CH-3	3.72		73.8		
	CH-4	3.42		70.7		
	CH-5	3.84		72.5		
	CH_2_-6	3.84; 3.78		61.6		
β-Glucose (β-Glc)	CH-1	4.65 *	d (8.0)	96.9	L, S	L, S, F
	CH-2	3.26		75.2		
	CH-3	3.50		76.7		
	CH-4	3.42		70.7		
	CH-5	3.47		76.9		
	CH_2_-6	3.90; 3.74		61.7		
α-Fructofuranose	CH-3	4.13 *		82.9	L, S	L, S, F
	CH-5	4.07		82.4		
β-Fructofuranose	CH_2_-1,1′	3.60; 3.57		63.8	L, S	L, S, F
	CH-3	4.12 *		76.4		
	CH-4	4.12 *		75.4		
	CH-5	3.83		81.6		
	CH_2_-6,6′	3.81; 3.68		63.3		
β-Fructopyranose	CH_2_-1, 1′	3.72; 3.56		64.8	L, S	L, S, F
	CH-3	3.81		68.5		
	CH-4	3.90		70.6		
	CH-5	4.00		70.2		
	CH_2_-6,6′	4.03; 3.71		64.4		
Sucrose (Suc)	CH-1	5.42 *	d (3.8)	93.2	L, S	L, S, F
	CH-2	3.56		72.0		
	CH-3	3.77		73.5		
	CH-4	3.48		70.2		
	CH-5	3.85		73.4		
	CH_2_-6	3.82		61.2		
	CH_2_-1′	3.69		62.4		
	C-2	/		104.8		
	CH-3′	4.22		77.4		
	CH-4′	4.06		75.0		
	CH-5′	3.90		82.4		
	CH-6′	3.82		63.4		
Raffinose	CH-1 (Gal)	5.00 *	d (3.8)	99.4		L, S
	CH-1(Glc)	5.44	d (3.8)	93.2		
Inulin	CH-1 (Glc)	5.44 *		93.5	L, S	
	CH-3 (Fru)	4.27		77.8		
	CH-4 (Fru)	4.10		75.3		
*chiro*-Inositol	CH-1,6	4.05		72.7	L, S	
	CH-2,5	3.76		71.4		
	CH-3,4	3.59 *		73.8		
*myo*-Inositol	CH-2,5	3.54		72.4	L, S	L, S, F
	CH-1	4.08		73.2		
	CH-3,6	3.63		73.5		
	CH-4	3.29 *		75.3		
*scyllo*-Inositol	CH-1,2,3,4,5,6	3.36 *	s	74.6	L, S	
	Glucosinolates					
Glucoiberin	CH-1′ (Gluc)	5.08 *	d (9.8)	82.7		L
	CH-2′	3.47		72.9		
	CH-3′	3.58		78.1		
	CH-4′	3.47		70.2		
	CH-5′	3.60		81.2		
	CH_2_-6a′,6b′	3.92; 3.73		61.7		
	S-CH_3_	2.74	s	37.7		
	α-CH_2_	3.05; 2.99		52.5		
	β-CH_2_	2.21		20.7		
	γ-CH_2_	2.95		31.9		
	C=N	-		163.6		
Glucobrassicin	CH-1′ (Gluc)	4.84		82.4		L
	CH-2′	3.31		72.8		
	CH-3′	3.23		77.9		
	CH-4′	3.38		69.6		
	CH-5′	2.96		80.9		
	CH_2_-6′	3.59		61.2		
	CH_2_-1a, 1b	4.28; 4.22		30.4		
	CH-2″ (Ind)	7.37		125.2		
	CH-4″	7.76 *	d (8.0)	119.5		
	CH-5″	7.21		120.8		
	CH-6″	7.28		123.2		
	CH-7″	7.56		113.1		
Glucoraphanin	CH-1′ (Gluc)	5.05	d (9.8)			L
	S-CH_3_	2.72	s			
Sinigrin	CH-1′ (Gluc)	5.07	d (9.8)			L
	Miscellaneous					
Chlorogenic acid (5-caffeoylquinic acid)	2,2′-CH_2_	2.19; 2.02		39.5	L, S	
	6,6′-CH_2_	2.14; 2.06		38.4		
	CH-3	4.26		71.7		
	CH-4	3.88		73.9		
	CH-5	5.32 *		72.2		
	CH-2′	7.18	d (1.6)	116.1		
	CH-5′	6.94	d (8.3)	117.5		
	CH-6′	7.09	dd (8.3; 1.6)	123.7		
	CH-7′	7.60	d (15.9)	147.2		
	CH-8′	6.37	d (15.9)	115.6		
Neochlorogenic acid (3-caffeoylquinic acid)	2,2′-CH_2_	2.21; 2.09			S	
	6,6′-CH_2_	2.11; 1.93				
	CH-3	5.40		74.0		
	CH-4	3.76				
	CH-5	4.17				
	CH-2′	7.23		116.1		
	CH-5′	6.97		117.5		
	CH-6′	7.14		123.7		
	CH-7′	7.66	d (16.0)	147.2		
	CH-8′	6.44	d (16.0)	115.6		
Glycine betaine	N(CH_3_)_3_	3.27 *		54.4	L	
	CH_2_	3.91		67.3		
Ethanolamine	CH_2_-NH_2_	3.15 *		42.2	L	L, S, F
	CH_2_OH	3.83		58.6		
Choline	N(CH_3_)_3_	3.21 *	s	54.9	L, S	L, S, F
	CH_2_OH	4.06		56.6		
	CH_2_N	3.52		68.4		
Phosphorylcholine	N(CH_3_)_3_	3.23 *	s	55.0	L, S	L, S, F
	CH_2_OPO_3_	4.15		62.2		
	CH_2_N	3.61		67.4		
Glycerophosphorylcholine	N(CH_3_)_3_	3.24 *	s	55.1	L	L, S, F
	CH_2_N	3.68		67.0		
	CH_2_OP	4.33		60.5		
	CH_2_OP	3.94; 3.89		67.5		
	CHOH	3.92		71.6		
	CH_2_OH	3.67; 3.6		63.1		
Trigonelline	CH_3_	4.44	s	49.3	L, S	L, F
	CH-6	9.12				
	CH-4,2	8.84 *				
	CH-3	8.09		128.8		
Uridine	CH-6	7.87	d (8.2)	143.0	L, S	L, S, F
	CH-5	5.92 *	d (8.2)	103.4		
	CH-1′ (rib)	5.93 *	d (4.6)	90.3		
	CH-2′ (rib)	4.36		74.6		
	CH-3′ (rib)	4.24		70.5		
	CH-4′ (rib)	4.14		85.3		

* Asterisks indicate signals used for the integration and quantification of metabolites.

**Table 2:** ^1^H- and ^13^C NMR data of sesquiterpene lactones in globe artichoke byproducts [CDCl_3_/CD_3_OD 2:1 *v*/*v*, ^1^H 600 MHz, ^13^C 150 MHz, ppm (*J* = Hz)]. Multiplicity: d = doublet; dd = doublet of doublets; ddd = doublet of doublets of doublets; dt = doublet of triplets; q = quartet; t = triplet.

Position	Cynaropicrin		Dehydrocynaropicrin		Grosheimin	
	^1^H	^13^C	^1^H	^13^C	^1^H	^13^C
1	2.99 ddd (10.2; 9.8; 7.2)	45.4	3.30	40.9	3.21	40.3
2a	2.17 dt (12.9; 7.1)	39.1	2.64 dd (18.6; 8.4)	43.7	2.58	43.7
2b	1.74 ddd (12.8;11.6; 8.6)	39.1	2.58	43.7	2.52	43.7
3	4.52 ddt (8.5; 7.3; 2.2)	73.3	-	205.2	-	220.8
4	-	152.3	-		2.34	47.5
5	2.87	51.3	3.35	49.4	2.35	51.3
6	4.31 dd (10.6; 9.0)	79.4	4.18 dd (9.9; 8.9)	80.4	4.03 t (9.0)	83.6
7	3.25	47.7	3.50	46.9	3.11	49.6
8	5.14 dd (5.1; 3.5)	74.5	5.13	74.5	3.80 dd (10.0; 6.1)	73.1
9a	2.72 dd (14.8; 5.2)	37.0	2.92 dd( 13.4; 5.7)	41.5	2.85	48.5
9b	2.41 dd (14.8; 3.6)	37.0	2.38	41.5	2.29	48.5
10	-	142.2	-		-	144.2
12	-	170.2	-	170.0	-	171.1
13a	6.20 d (3.5)	122.9	6.30 d (3.3)	125.0	6.37 dd (2.8; 1.2)	126.0
13b	5.67 * d (3.3)	122.9	5.85 * d (3.0)	125.0	6.33 * dd (3.3; 1.2)	126.0
14a	5.16	118.2	5.09	115.2	5.07	115.2
14b	4.94 d (1.5)	118.2	4.77	115.2	4.78	115.2
15a	5.47 t (1.8)	113.0	6.28 dd (2.4; 0.6)	123.6	1.26	14.9
15b	5.38 t (2.3)	113.0	5.89 dd (2.2; 0.6)	123.6		
1′	-	165.7	-			
2′	-	140.2	-			
3′a	6.35 q (1.2)	126.0	6.37	126.3		
3′b	6.01 q (1.6)	126.0	6.04	126.3		
4′	4.35 t (1.3)	61.0	4.36	61.0		

* Asterisks indicate signals used for integration and quantification of metabolites.

**Table 3:** Metabolites identified in chloroform extracts of cauliflower and globe artichoke byproducts. Relative assignments of ^1^H and ^13^C NMR signals are reported. L = leaves; S = stalks; F = florets; d = doublet; dd = doublet of doublets; s = singlet; t = triplet.

Metabolite	Assignment	δ ^1^H (ppm)	Multiplicity	δ ^13^C (ppm)	Globe Artichoke	Cauliflower
Pheophytin a	CH-10	9.54	s	105.0	L	L
	CH-5	9.39 *	s	97.8		
	CH-20	8.59	s	93.7		
	CH-3^1^	8.01	dd (17.7; 11.5)	129.3		
	CH_2_-3^2^	6.32; 6.22	dd (17.7; 1.1) dd (11.5; 1.2)	123.5		
	CH-P2	4.89		118.0		
	CH-18	4.48		50.5		
	CH-17	4.12		52.0		
	CH_3_-13^4^	3.91	s	53.1		
	CH_2_-8^1^	3.70		19.7		
	CH_3_-18^1^	1.82	d (7.6)	23.4		
	CH_3_-8^2^	1.71	t (7.9)	17.6		
Pheophytin b	CH-7^1^	11.19	s		L	L
	CH-5	9.98 *	s			
	CH-10	9.65	s			
	CH-3^1^	7.93	dd (17.8; 11.5)			
	CH_2_-3^2^	6.24; 6.02				
Squalene	CH_3_ -a	1.69		25.3	L, S	L, F
	CH_3_ -b	1.61		16.2		
	CH -c	5.12 *		124.6		
	CH_2_-d	1.99		40.2		
	CH_2_-e	2.07		26.8		
Linolenic acid chains	CH_2_-2	2.32		34.6	L, S	L, S, F
	CH_2_-3	1.63		25.3		
	CH_2_-4-7	1.32				
	CH_2_-8	2.06		27.5		
	CH-9	5.38		130.3		
	CH_2_-11,14	2.81 *	t (6.1)	25.9		
	CH-10, 12,13	5.36		128.5		
	CH-15	5.31		127.4		
	CH-16	5.39		132.2		
	CH_2_-17	2.09		20.9		
	CH_3_-18	0.98	t (7.6)	14.5		
Linoleic acid chains	CH_2_-2	2.32		34.6	L, S	L, S, F
	CH_2_-3	1.63		25.3		
	CH_2_-4–7	1.32				
	CH_2_-8,14	2.06		27.5		
	CH-9,13	5.37		130.5		
	CH-10,12	5.35		128.4		
	CH_2_-11	2.78 *	t (6.7)	25.8		
	CH_3_-18	0.90	t (7.6)	14.3		
Phosphatidylcholine	(CH_3_)_3_N	3.23		54.5	L, S	L, F, S
	CH_2_OP	4.44		61.9		
	CH_2_ sn1	4.39; 4.17		63.1		
	CH sn2	5.26		70.8		
	CH_2_ sn3	4.14		65.4		
Phosphatidylethanolamine	CH_2_NH_2_	3.16		40.8		S, F
	CH_2_OP	4.10		62.1		
Digalactosyldiacylglycerol	CH-1′	4.23		104.3	L, S	L, F, S
	CH-2′	3.53		71.7		
	CH-3′	3.51		73.8		
	CH-4′	3.92		68.5		
	CH-1″	4.91 *		99.7		
	CH-3″,5″	3.74		70.6		
	CH-4″	3.96		70.2		
	CH_2_-6″	3.82; 3.74		61.8		
	CH_2_ sn3	3.95; 3.72		68.5		
	CH_2_ sn1	4.39; 4.17		63.1		
β-Sitosterol	CH_2_-1	1.85; 1.07		37.6	L, S	L, F, S
	CH_2_-2	1.82		31.4		
	CH-3	3.54		71.6		
	CH_2_-4	2.25		42.1		
	CH-6	5.34		121.8		
	CH-8	1.47		32.2		
	CH-9	0.94		50.6		
	CH_2_-11	1.51		21.4		
	CH_2_-12	2.00; 1.17		40.0		
	CH-14	1.01		57.1		
	CH_2_-16	1.86		28.4		
	CH-17	1.13		56.4		
	CH_3_-18	0.70 *		12.1		
	CH_3_-19	1.02		19.5		
	CH-20	1.35		36.5		
	CH_3_-21	0.94		18.9		
	CH_2_-23	1.19		26.4		
	CH-24	0.95		46.1		
Campesterol	CH_3_-18	0.70 *		12.0	L, S	L, F, S
Stigmasterol	CH_3_-18	0.72 *		11.9	L, S	

* Asterisks indicate signals used for integration and quantification of metabolites.

**Table 4:** Metabolite content (in mg/g DW) in aqueous extracts of cauliflower and globe artichoke byproducts.

Metabolite	Globe Artichoke		Cauliflower		
	L	S	L	S	F
	Mean ± SD	Mean ± SD	Mean ± SD	Mean ± SD	Mean ± SD
Amino acids					
Alanine	0.30 ± 0.017 ^a^	0.29 ± 0.015 ^a^	4.72 ± 0.22 ^a^	1.96 ± 0.02 ^b^	7.01 ± 0.05 ^c^
Arginine			5.23 ± 0.22		3.90 ± 0.45
Asparagine	14.48 ± 1.45 ^a^	2.59 ± 0.43 ^b^	4.04 ± 0.27 ^a^	2.78 ± 0.05 ^b^	6.83 ± 0.13 ^c^
Aspartate	1.11 ± 0.19 ^a^	0.28 ± 0.036 ^b^	2.41 ± 0.23 ^a^	2.66 ± 0.30 ^a^	6.03 ± 0.14 ^b^
GABA			4.27 ± 0.14 ^a^	1.18 ± 0.12 ^b^	4.68 ± 0.20 ^c^
Glutamate	1.41 ± 0.12 ^a^	0.55 ± 0.058 ^b^	2.76 ± 0.09 ^a^	1.97 ± 0.17 ^b^	3.86 ± 0.07 ^c^
Glutamine	1.52 ± 0.065 ^a^	0.70 ± 0.264 ^b^	9.78 ± 0.64 ^a^	12.19 ± 0.45 ^b^	37.63 ± 0.54 ^c^
Histidine	0.27 ± 0.093 ^a^	0.039 ± 0.011 ^b^	1.06 ± 0.02 ^a^	0.21 ± 0.019 ^b^	1.09 ± 0.01 ^a^
Isoleucine	0.12 ± 0.013 ^a^	0.058 ± 0.0061 ^b^	1.23 ± 0.04 ^a^	0.32 ± 0.024 ^b^	1.17 ± 0.03 ^a^
Leucine	0.20 ± 0.009 ^a^	0.064 ± 0.0092 ^b^	1.46 ± 0.08 ^a^	0.19 ± 0.008 ^b^	0.58 ± 0.005 ^c^
Lysine			1.41 ± 0.08		0.41 ± 0.013
Methiin			4.83 ± 0.04 ^a^	4.95 ± 0.27 ^a^	20.71 ± 0.24 ^b^
Phenylalanine	1.32 ± 0.071 ^a^	0.17 ± 0.013 ^b^	2.24 ± 0.10 ^a^	0.23 ± 0.012 ^b^	0.84 ± 0.016 ^c^
Threonine	0.49 ± 0.030 ^a^	0.14 ± 0.012 ^b^	2.37 ± 0.11 ^a^	1.19 ± 0.09 ^b^	1.76 ± 0.09 ^c^
Tryptophan	0.56 ± 0.028		0.66 ± 0.05 ^a^	0.12 ± 0.012 ^b^	0.26 ± 0.004 ^c^
Tyrosine			1.20 ± 0.08 ^a^	0.14 ± 0.010 ^b^	0.37 ± 0.007 ^c^
Valine	0.50 ± 0.018 ^a^	0.15 ± 0.020 ^b^	3.09 ± 0.01 ^a^	1.01 ± 0.021 ^b^	3.94 ± 0.03 ^c^
Organic acids					
Acetic acid	0.14 ± 0.049 ^a^	0.061 ± 0.011 ^a^	2.68 ± 0.209 ^a^	0.10 ± 0.031 ^b^	0.36 ± 0.032 ^b^
Citric acid	5.30 ± 0.192 ^a^	0.58 ± 0.026 ^b^	5.80 ± 0.03 ^a^	6.67 ± 0.06 ^b^	5.95 ± 0.27 ^a^
Formic acid	0.034 ± 0.009 ^a^	0.032 ± 0.006 ^a^	0.051 ± 0.006 ^a^	0.029 ± 0.011 ^b^	0.027 ± 0.001 ^b^
Fumaric acid	0.082 ± 0.011 ^a^	0.051 ± 0.010 ^b^	0.045 ± 0.002 ^a^	0.082 ± 0.024 ^b^	1.04 ± 0.005 ^c^
Lactic acid		0.34 ± 0.085	0.73 ± 0.086 ^a^	0.040 ± 0.009 ^b^	0.065 ± 0.011 ^b^
Malic acid	7.48 ± 0.54 ^a^	8.32 ± 0.77 ^a^	3.32 ± 0.24 ^a^	12.90 ± 0.18 ^b^	24.72 ± 0.14 ^c^
Quinic acid	6.51 ± 0.57 ^a^	7.61 ± 0.26 ^b^			
Succinic acid	1.97 ± 0.05 ^a^	1.15 ± 0.12 ^b^	2.87 ± 0.381	0.505 ± 0.014	
Carbohydrates, polyols					
Fructose	5.15 ± 1.07 ^a^	30.2 ± 0.35 ^b^	37.90 ± 4.33 ^a^	83.08 ± 2.23 ^b^	68.33 ± 0.39 ^c^
Glucose	24.8 ± 5.00 ^a^	203.5 ± 4.02 ^b^	61.07 ± 1.66 ^a^	196.8 ± 6.88 ^b^	70.72 ± 0.43 ^a^
Inulin	0.44 ± 0.026 ^a^	4.14 ± 0.65 ^b^			
Raffinose			0.69 ± 0.032	3.09 ± 0.27	
Sucrose	5.87 ± 1.56 ^a^	29.47 ± 3.10 ^b^	6.68 ± 0.62 ^a^	27.32 ± 0.47 ^b^	21.22 ± 0.44 ^c^
*chiro*-Inositol	10.07 ± 2.69 ^a^	5.05 ± 0.33 ^b^			
*myo*-Inositol	0.27 ± 0.013 ^a^	1.95 ± 0.15 ^b^	3.06 ± 0.20	2.61 ± 0.38	
*scyllo*-Inositol	1.78 ± 0.05 ^a^	0.57 ± 0.034 ^b^			
*Miscellaneous*					
Glucoiberin			2.09 ± 0.138		
Glucobrassicin			1.43 ± 0.078		
Chlorogenic acid	7.53 ± 0.41 ^a^	2.32 ± 0.26 ^b^			
Glycine betaine	0.30 ± 0.029 ^a^				
Choline	1.01 ± 0.09 ^a^	0.35 ± 0.005 ^b^	1.57 ± 0.077 ^a^	0.73 ± 0.022 ^b^	4.73 ± 0.06 ^c^
Ethanolamine	0.066 ± 0.010 ^a^	0.11 ± 0.010 ^b^	0.31 ± 0.017 ^a^	0.22 ± 0.015 ^b^	0.79 ± 0.012 ^c^
Glycerophosphorylcholine	0.090 ± 0.007 ^a^			0.42 ± 0.059	
Phosphorylcholine	0.28 ± 0.039 ^a^	0.12 ± 0.005 ^b^	0.11 ± 0.016 ^a^	0.25 ± 0.024 ^b^	0.58 ± 0.029 ^c^
Trigonelline	0.30 ± 0.012 ^a^	0.064 ± 0.003 ^b^			0.063 ± 0.003
Uridine	0.13 ± 0.010 ^a^	0.057 ± 0.010 ^b^	0.373 ± 0.040 ^a^	0.14 ± 0.005 ^b^	0.55 ± 0.006 ^c^

^a,b,c^ The different superscript letters indicate the significant difference between mean values (*p* < 0.05) according to ANOVA.

**Table 5:** Metabolite content (in µmol/g DW) in organic extracts of cauliflower and globe artichoke byproducts. Sesquiterpene lactones content is reported in mg/g DW.

Metabolite	Globe Artichoke		Cauliflower		
	L	S	L	S	F
	Mean ± SD	Mean ± SD	Mean ± SD	Mean ± SD	Mean ± SD
Sterols					
β-Sitosterol + Campesterol	7.53 ± 0.68 ^b^	4.43 ± 0.27 ^a^	9.07 ± 1.26 ^a^	7.14 ± 1.91 ^a^	25.53 ± 2.13 ^b^
Stigmasterol	2.56 ± 0.25 ^b^	1.26 ± 0.10 ^a^			
Fatty acids					
Saturated + monounsaturated fatty chains	65.1 ± 14.8 ^a^	73.3 ± 11.2 ^a^	61.5 ± 15.5 ^a^	52.5 ± 21.8 ^a^	109.3 ± 1.8 ^b^
Linoleic fatty chains	18.9 ± 1.5 ^b^	12.9 ± 0.4 ^a^	22.2 ± 3.6 ^b^	8.0 ± 1.5 ^a^	29.3 ± 2.4 ^c^
Linolenic fatty chains	58.0 ± 4.3 ^b^	8.2 ± 0.5 ^a^	76.7 ± 10.8 ^b^	17.2 ± 0.6 ^a^	104.7 ± 8.1 ^c^
Miscellaneous					
Digalactosyldiacylglycerol	7.55 ± 0.63 ^b^	1.52 ± 0.14 ^a^	5.41 ± 0.72 ^c^	0.80 ± 0.19 ^a^	2.36 ± 0.26 ^b^
Squalene	9.07 ± 2.24 ^b^	1.35 ± 0.27 ^a^	6.83 ± 0.93		1.61 ± 0.15
Pheophytin a	4.76 ± 0.97		4.68 ± 1.53		
Pheophytin b	1.30 ± 0.24		0.86 ± 0.14		
*Sesquiterpene lactones*					
Cynaropicrin	27.5 ± 2.7 ^b^	2.47 ± 0.21 ^a^			
Dehydrocynaropicrin	7.22 ± 0.92				
Grosheimin	10.8 ± 1.7 ^b^	1.00 ± 0.10 ^a^			

^a,b,c^ The different superscript letters indicate the significant difference between mean values (*p* < 0.05) according to ANOVA.

## Data Availability

The data presented in this study are available in the article and in the supplementary material.

## Supplementary Materials

The following supporting information can be downloaded at: https://www.mdpi.com/article/10.3390/molecules28031363/s1; Figure S1: ^1^H NMR spectra of aqueous extract from globe artichoke byproducts: stalks (a) and leaves (b); Figure S2: The ^1^H-^13^C HSQC NMR 2D map of globe artichoke leaves aqueous extract; Figure S3: The ^1^H-^13^C HMBC NMR 2D map of globe artichoke leaves aqueous extract; Figure S4: The ^1^H-^1^H TOCSY NMR 2D map of globe artichoke leaves aqueous extract; Figure S5: The ^1^H-^13^C HSQC NMR 2D map of globe artichoke stalks aqueous extract; Figure S6: The ^1^H-^13^C HMBC NMR 2D map of globe artichoke stalks aqueous extract; Figure S7: The ^1^H-^1^H TOCSY NMR 2D map of globe artichoke stalks aqueous extract; Figure S8: The 3.64–3.52 ppm ^1^H NMR spectral region of globe artichoke leaves aqueous extract (blue) in comparison with fructose ^1^H NMR spectrum (red trace). Asterisks indicate the components of the *chiro*-inositol multiplet signal; Figure S9: The ^1^H-^13^C HSQC NMR 2D map of globe artichoke leaves chloroform extract; Figure S10: The ^1^H-^13^C HMBC NMR 2D map of globe artichoke leaves chloroform extract; Figure S11: The ^1^H-^1^H TOCSY NMR 2D map of globe artichoke leaves chloroform extract; Figure S12: The ^1^H-^13^C HSQC NMR 2D map of globe artichoke stalks chloroform extract; Figure S13: The ^1^H-^13^C HMBC NMR 2D map of globe artichoke stalks chloroform extract; Figure S14: The ^1^H-^1^H TOCSY NMR 2D map of globe artichoke stalks chloroform extract; Figure S15: The ^1^H-^13^C HSQC NMR 2D map of cauliflower leaves aqueous extract; Figure S16: The ^1^H-^13^C HMBC NMR 2D map of cauliflower leaves aqueous extract; Figure S17: The ^1^H-^1^H TOCSY NMR 2D map of cauliflower leaves aqueous extract; Figure S18: The ^1^H-^13^C HSQC NMR 2D map of cauliflower florets aqueous extract; Figure S19: The ^1^H-^13^C HMBC NMR 2D map of cauliflower florets aqueous extract; Figure S20: The ^1^H-^1^H TOCSY NMR 2D map of cauliflower florets aqueous extract; Figure S21: The ^1^H-^13^C HSQC NMR 2D map of cauliflower stalks aqueous extract; Figure S22: The ^1^H-^13^C HMBC NMR 2D map of cauliflower stalks aqueous extract; Figure S23: The ^1^H-^1^H TOCSY NMR 2D map of cauliflower stalks aqueous extract; Figure S24: ^1^H NMR spectra of cauliflower florets (a), stalks (b) and leaves (c) chloroform extracts. Solvent: CDCl_3_/CD_3_OD 2:1 *v/v*; Figure S25: The ^1^H-^13^C HSQC NMR 2D map of cauliflower leaves chloroform extract; Figure S26: The ^1^H-^13^C HMBC NMR 2D map of cauliflower leaves chloroform extract; Figure S27: The ^1^H-^1^H TOCSY NMR 2D map of cauliflower leaves chloroform extract; Figure S28: The ^1^H-^13^C HSQC NMR 2D map of cauliflower florets chloroform extract; Figure S29: The ^1^H-^13^C HMBC NMR 2D map of cauliflower florets chloroform extract; Figure S30: The ^1^H-^1^H TOCSY NMR 2D map of cauliflower florets chloroform extract; Figure S31: The ^1^H-^13^C HSQC NMR 2D map of cauliflower stalks chloroform extract; Figure S32: The ^1^H-^13^C HMBC NMR 2D map of cauliflower stalks chloroform extract; Figure S33: The ^1^H-^1^H TOCSY NMR 2D map of cauliflower stalks chloroform extract.
