# Supplementary material for: Byproducts of Globe Artichoke and Cauliflower Production as a New Source of Bioactive Compounds in the Green Economy Perspective: An NMR Study
Source: Molecules. 2023 Jan 31;28(3):1363. doi: 10.3390/molecules28031363 (PMC9919138; doi:10.3390/molecules28031363)
Supplement: Supplementary file 1 [file molecules-28-01363-s001.zip › molecules-2143642-supplementary.pdf]

Supplementary Materials

Byproducts of Globe Artichoke and Cauliflower Production as a New Source of Bioactive Compounds in the Green Economy Perspective: An NMR Study

Cinzia Ingallina [1,†], Giacomo Di Matteo [1,†], Mattia Spano [1], Erica Acciaro [2], Enio Campiglia [3], Luisa Mannina [1] and Anatoly Petrovich Sobolev [2,*]

[1] Department of Chemistry and Technology of Drugs, Food Chemistry Lab, Sapienza University of Rome, P.le Aldo Moro 5, 00185 Rome, Italy

[2] "Annalaura Segre" Magnetic Resonance Laboratory, Institute for Biological Systems, CNR, Via Salaria, Km 29,300, 00015 Monterotondo, Italy

[3] Department of Agricultural and Forest Sciences, University of Tuscia, Via San Camillo de Lellis, snc, 01100 Viterbo, Italy

* Correspondence: anatoly.sobolev@cnr.it
† These authors contributed equally to this work.

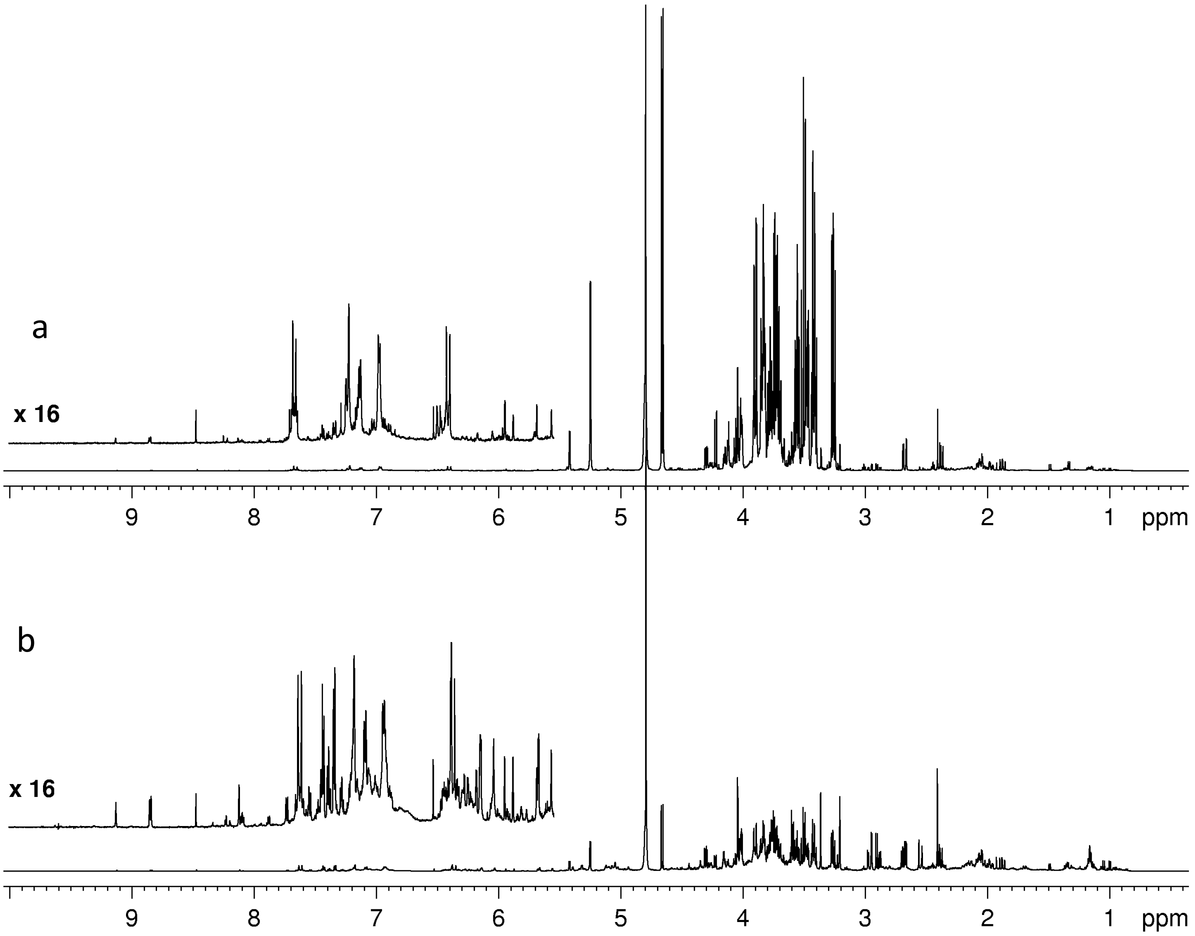

Figure S1. ^{1}H NMR spectra of aqueous extract from globe artichoke byproducts: stalks (a) and leaves (b).

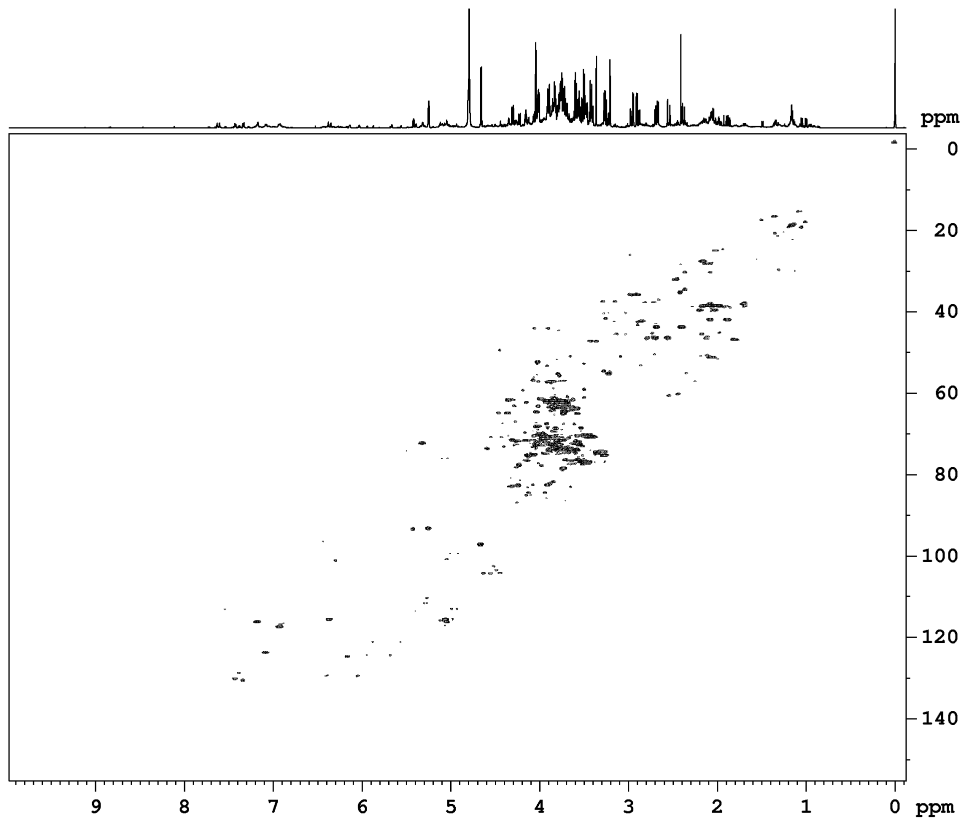

Figure S2. The ^{1}H-^{13}C HSQC NMR 2D map of globe artichoke leaves aqueous extract.

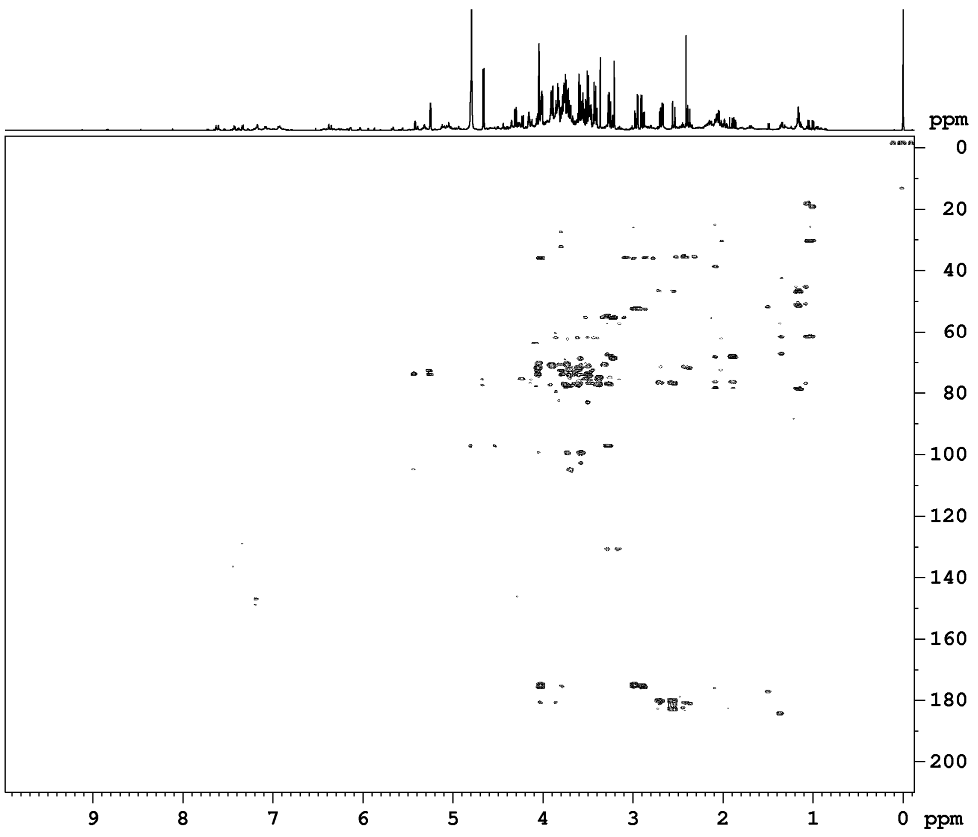

Figure S3. The ^{1}H-^{13}C HMBC NMR 2D map of globe artichoke leaves aqueous extract.

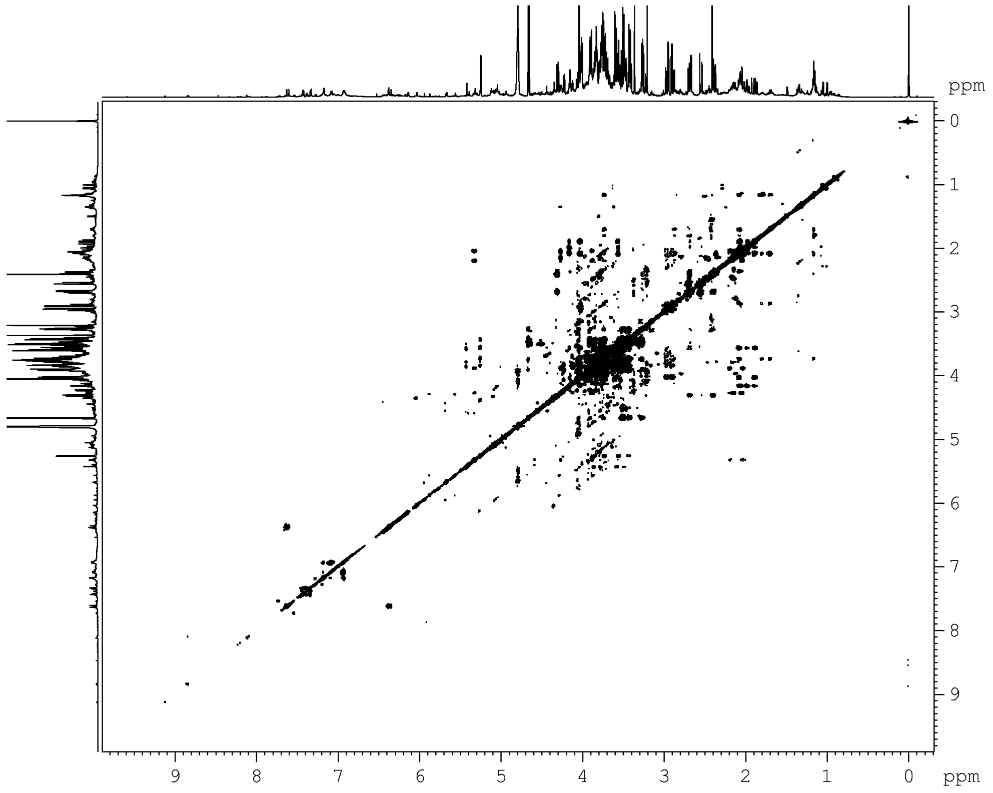

Figure S4. The ^{1}H-^{1}H TOCSY NMR 2D map of globe artichoke leaves aqueous extract.

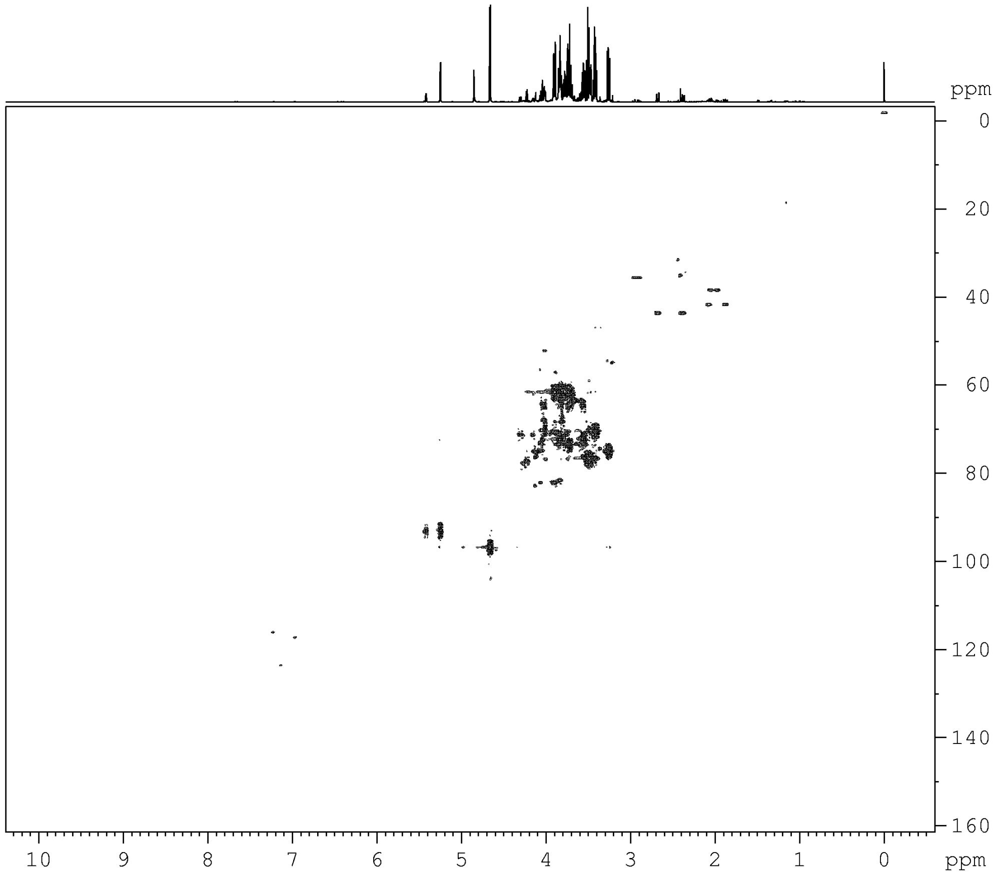

Figure S5. The ^{1}H-^{13}C HSQC NMR 2D map of globe artichoke stalks aqueous extract.

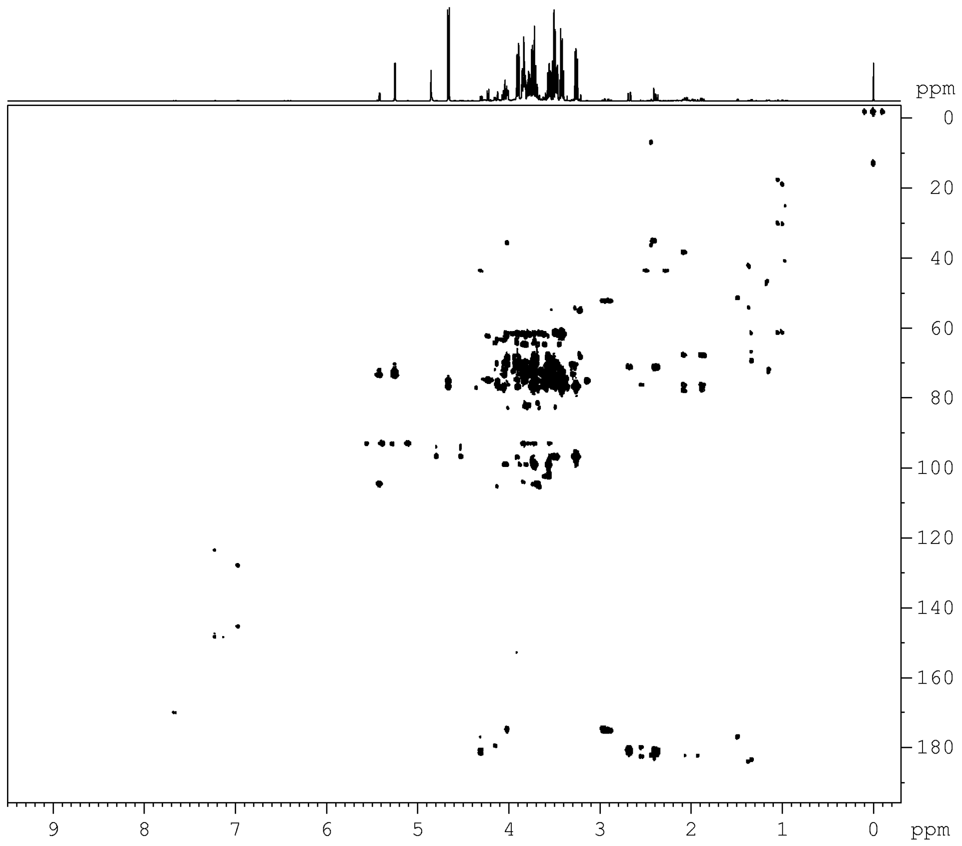

Figure S6. The ^{1}H-^{13}C HMBC NMR 2D map of globe artichoke stalks aqueous extract.

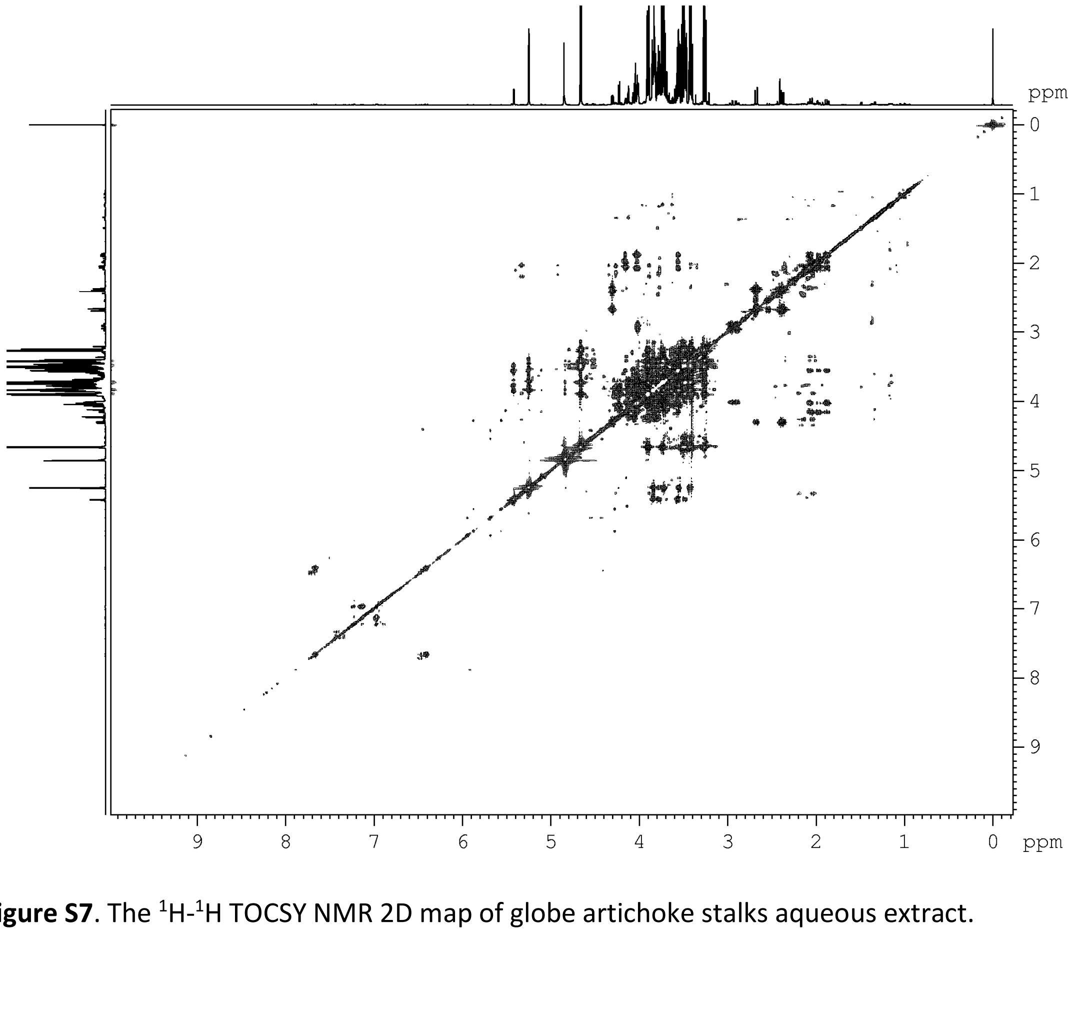

Figure S7. The ^{1}H-^{1}H TOCSY NMR 2D map of globe artichoke stalks aqueous extract.

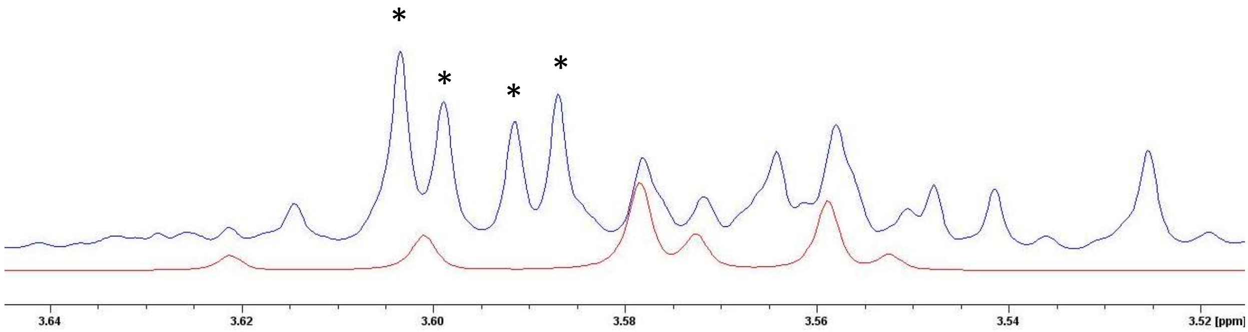

Figure S8. The 3.64 -3.52 ppm ^{1}H NMR spectral region of globe artichoke leaves aqueous extract (blue) in comparison with fructose ^{1}H NMR spectrum (red trace). Asterisks indicate the components of the *chiro*-inositol multiplet signal.

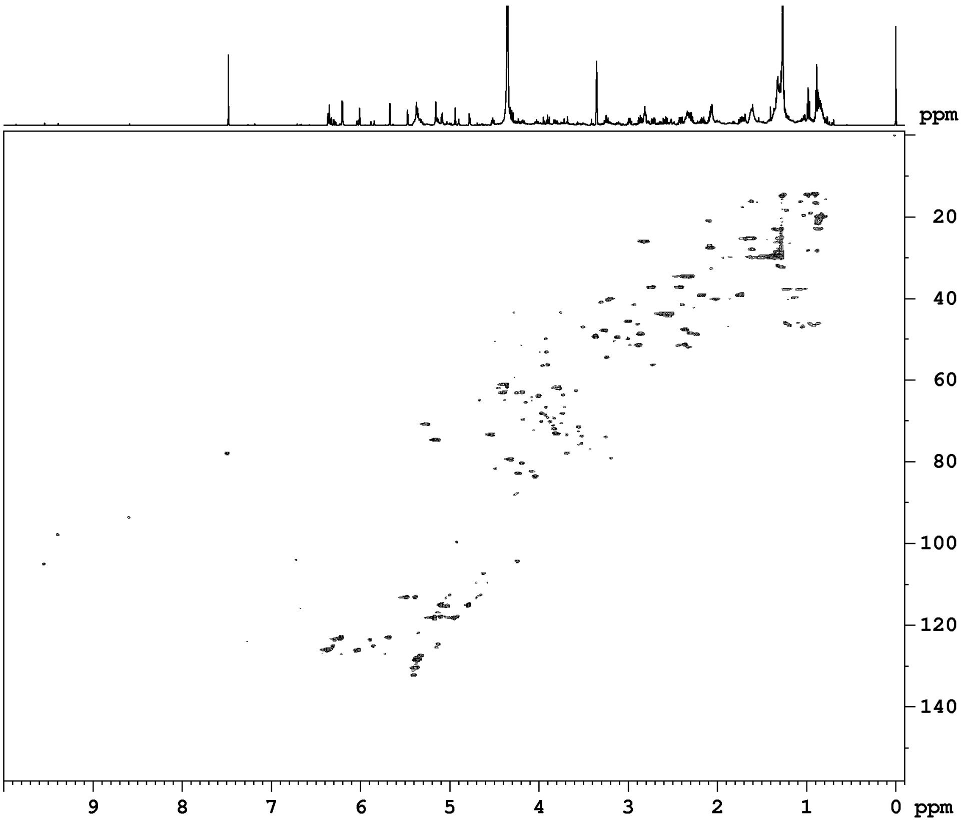

Figure S9. The ^{1}H-^{13}C HSQC NMR 2D map of globe artichoke leaves chloroform extract.

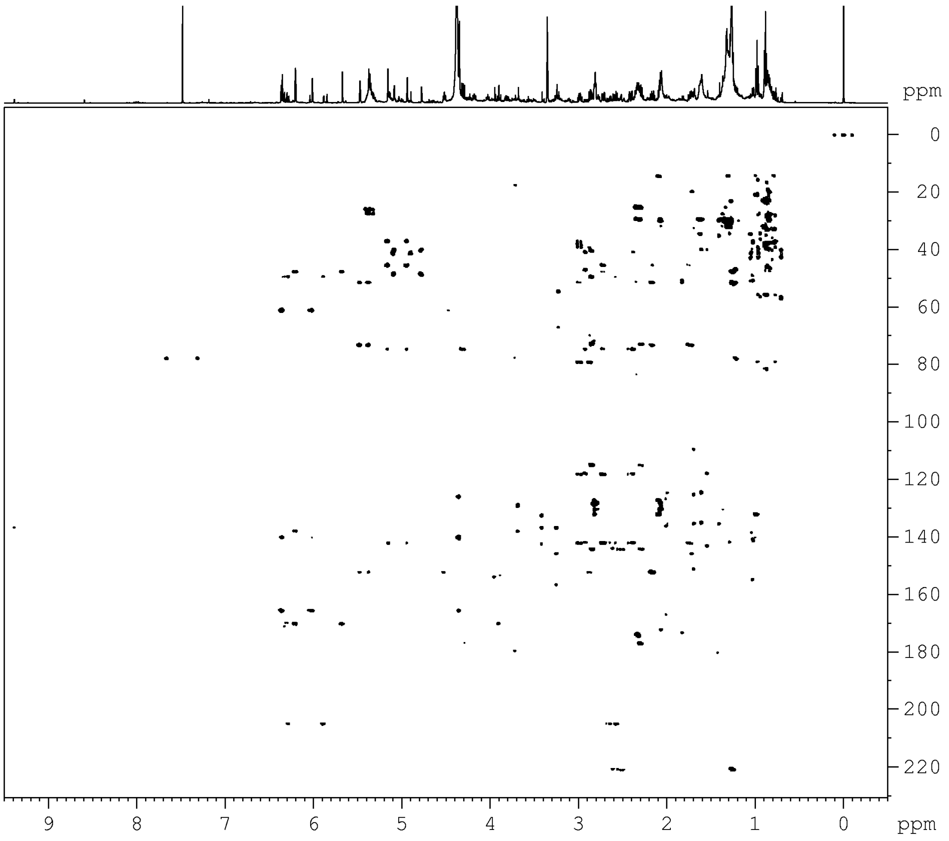

Figure S10. The ^{1}H-^{13}C HMBC NMR 2D map of globe artichoke leaves chloroform extract.

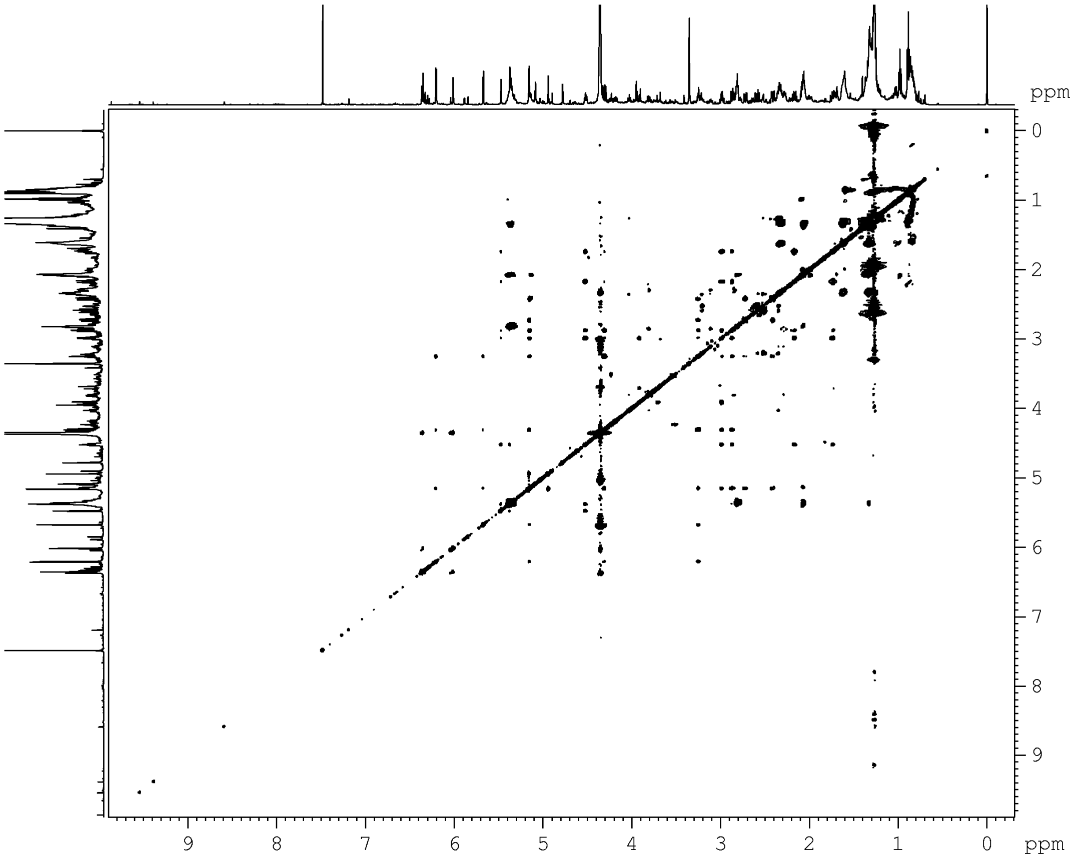

Figure S11. The ^{1}H-^{1}H TOCSY NMR 2D map of globe artichoke leaves chloroform extract.

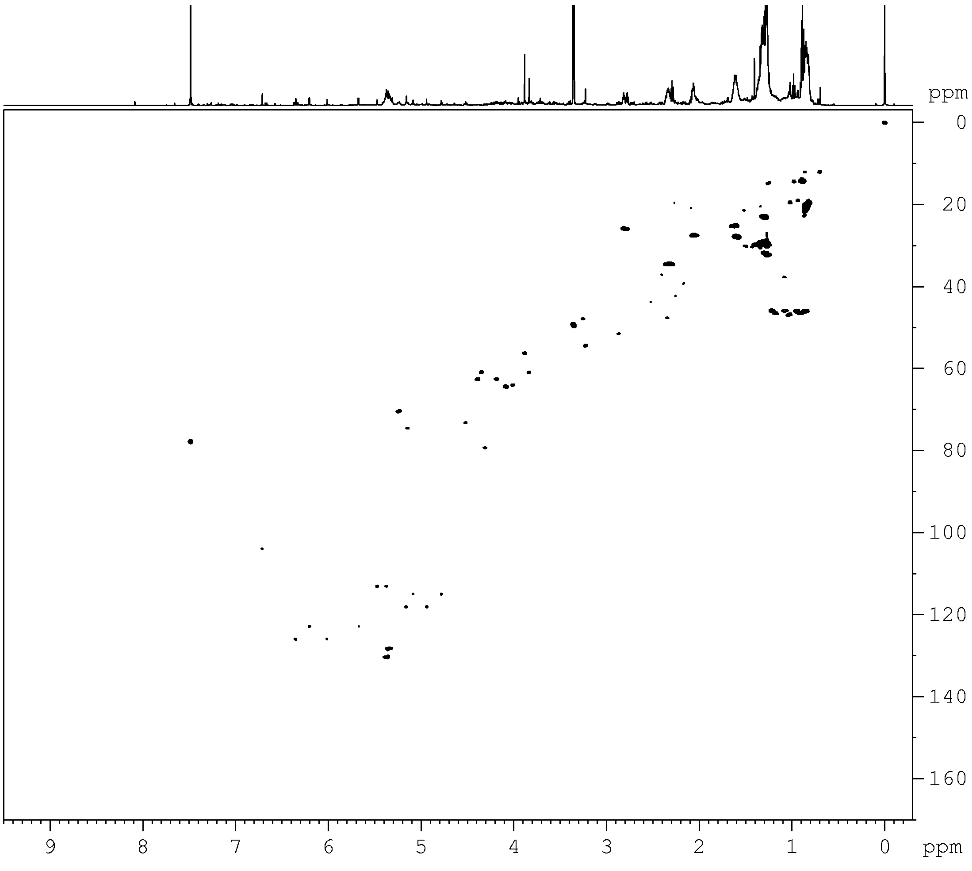

Figure S12. The ^{1}H-^{13}C HSQC NMR 2D map of globe artichoke stalks chloroform extract.

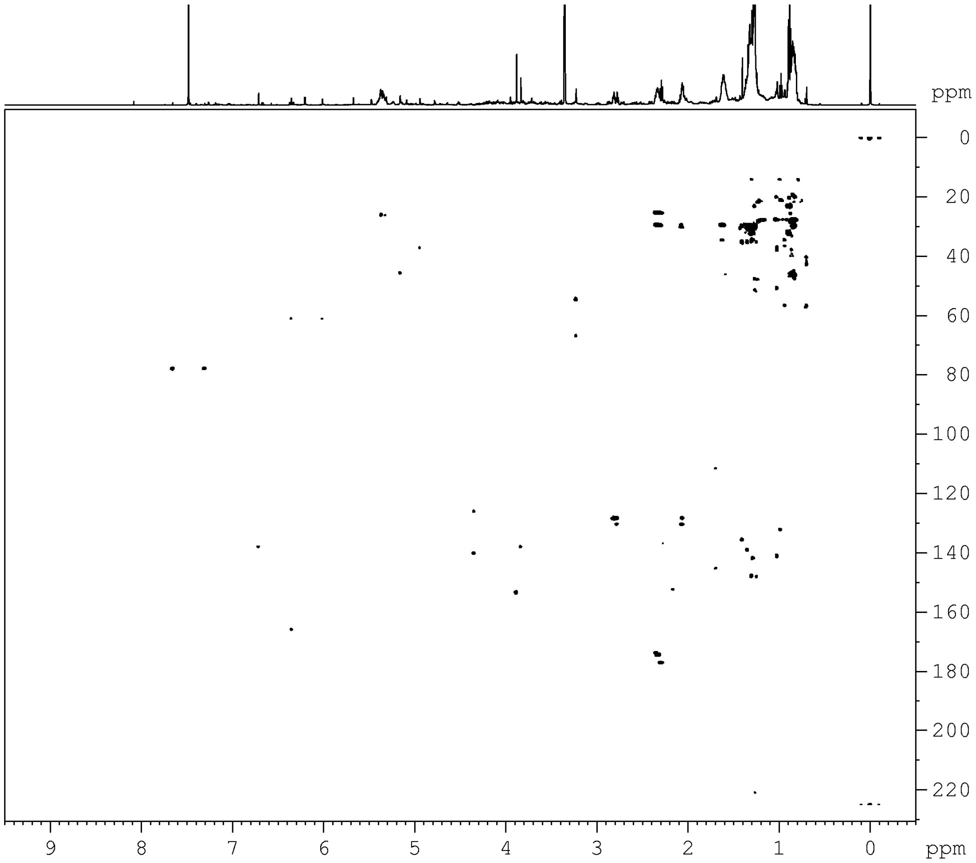

Figure S13. The ^{1}H-^{13}C HMBC NMR 2D map of globe artichoke stalks chloroform extract.

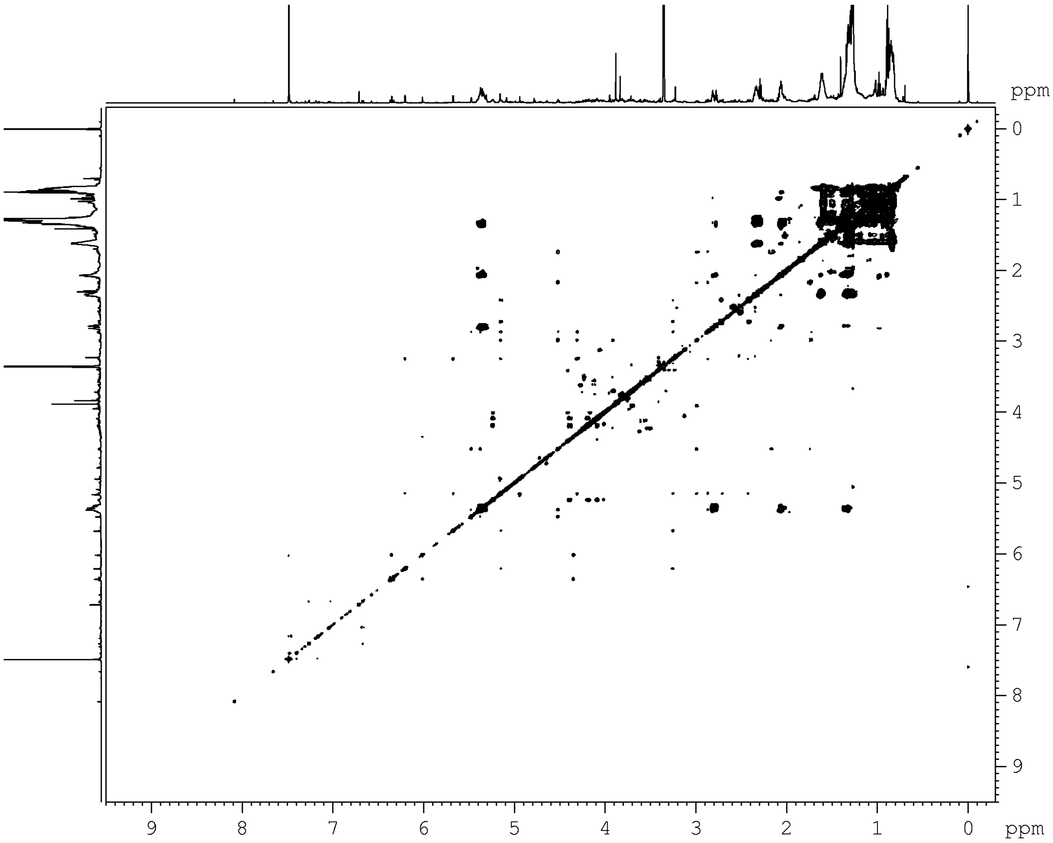

Figure S14. The ^{1}H-^{1}H TOCSY NMR 2D map of globe artichoke stalks chloroform extract.

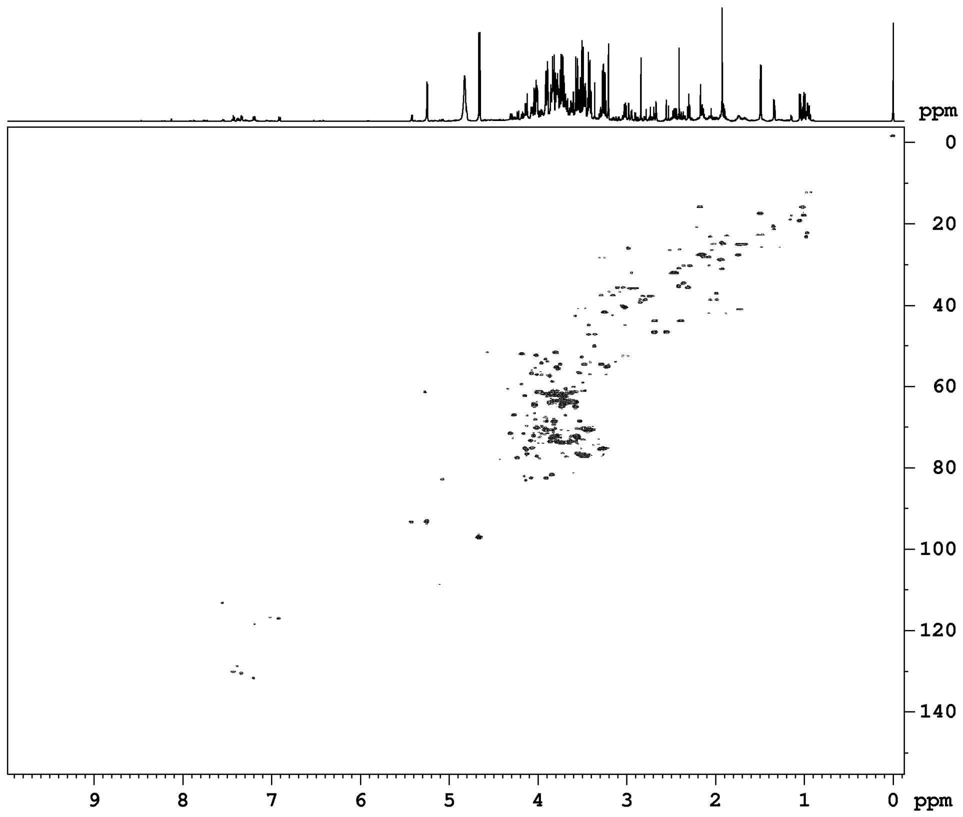

Figure S15. The ^{1}H-^{13}C HSQC NMR 2D map of cauliflower leaves aqueous extract.

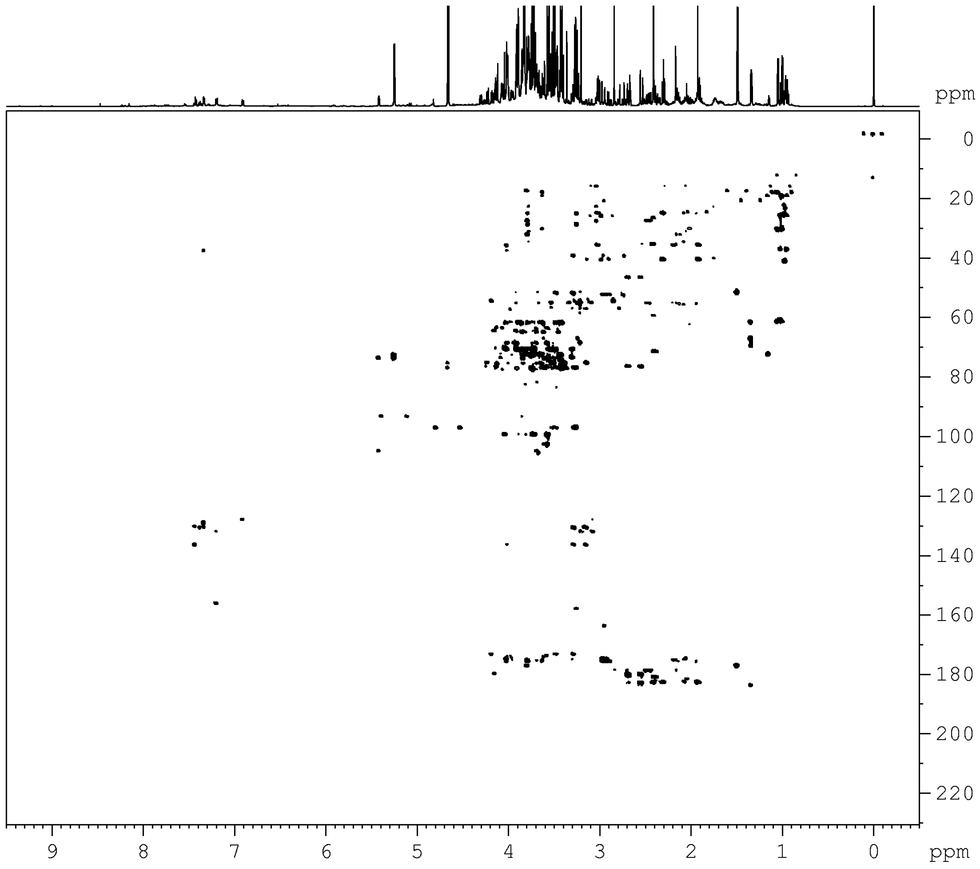

Figure S16. The ^{1}H-^{13}C HMBC NMR 2D map of cauliflower leaves aqueous extract.

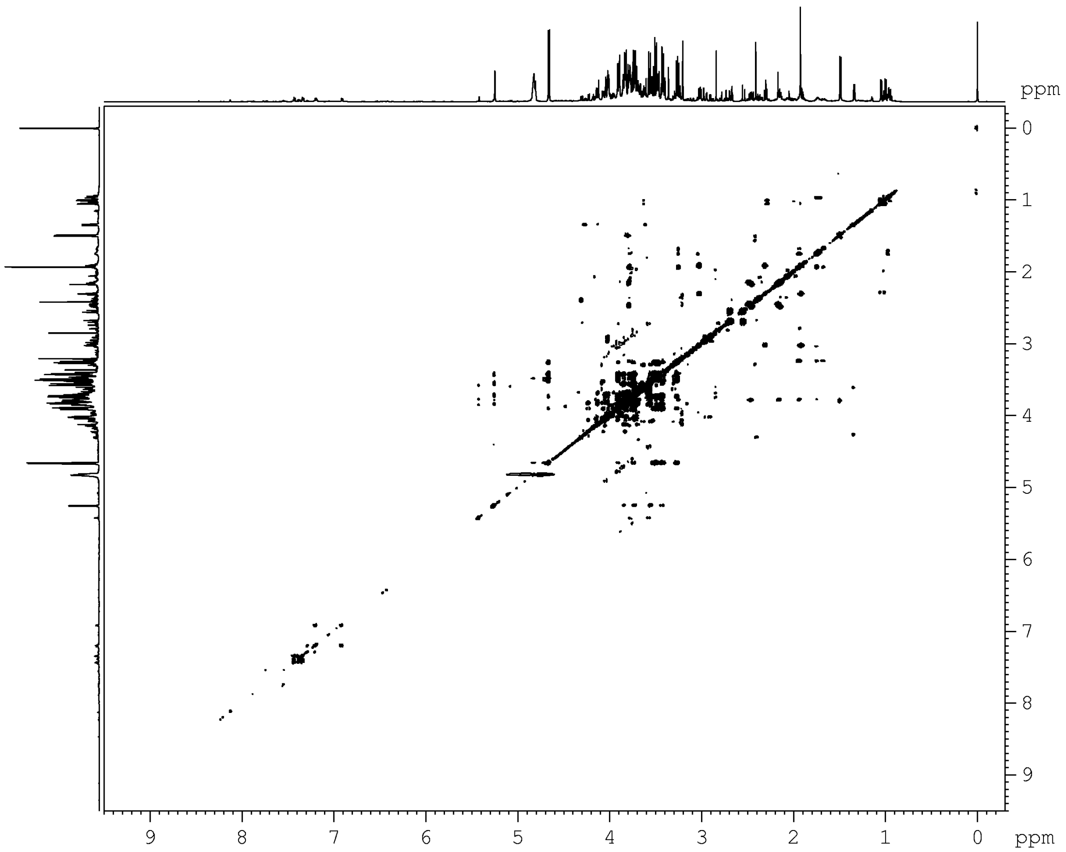

Figure S17. The ^{1}H-^{1}H TOCSY NMR 2D map of cauliflower leaves aqueous extract.

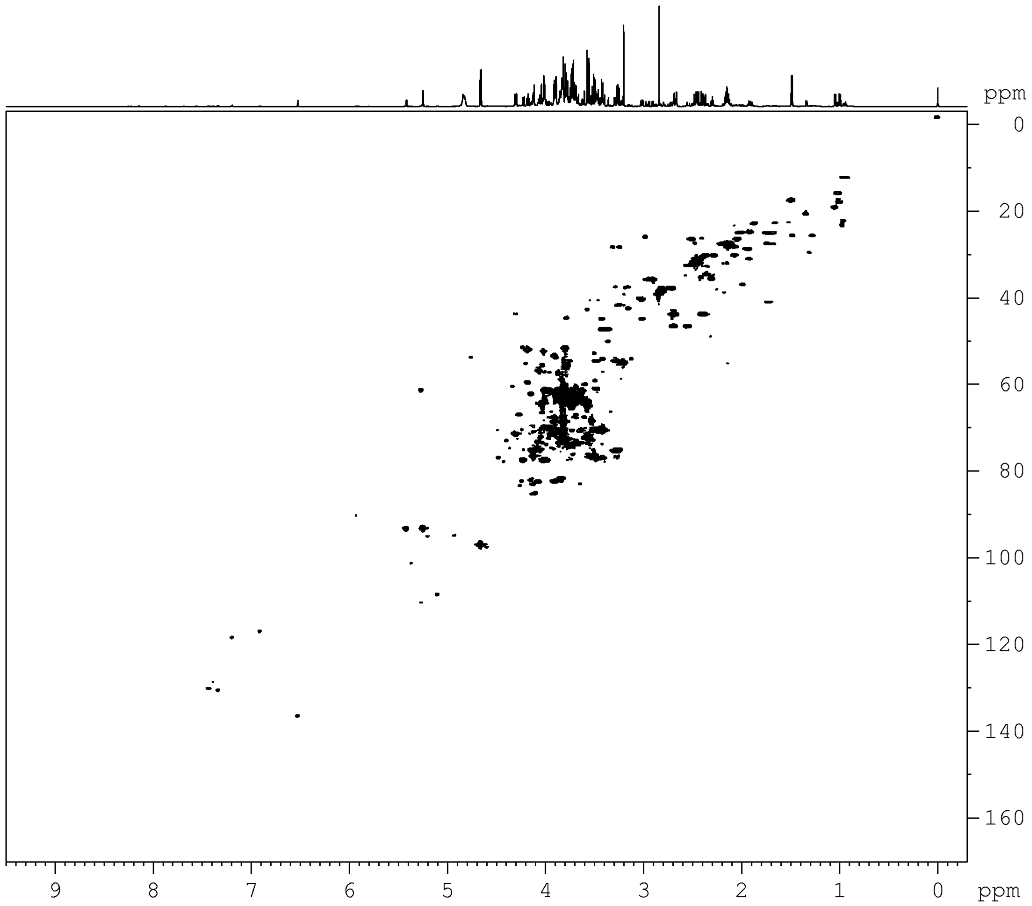

Figure S18. The ^{1}H-^{13}C HSQC NMR 2D map of cauliflower florets aqueous extract.

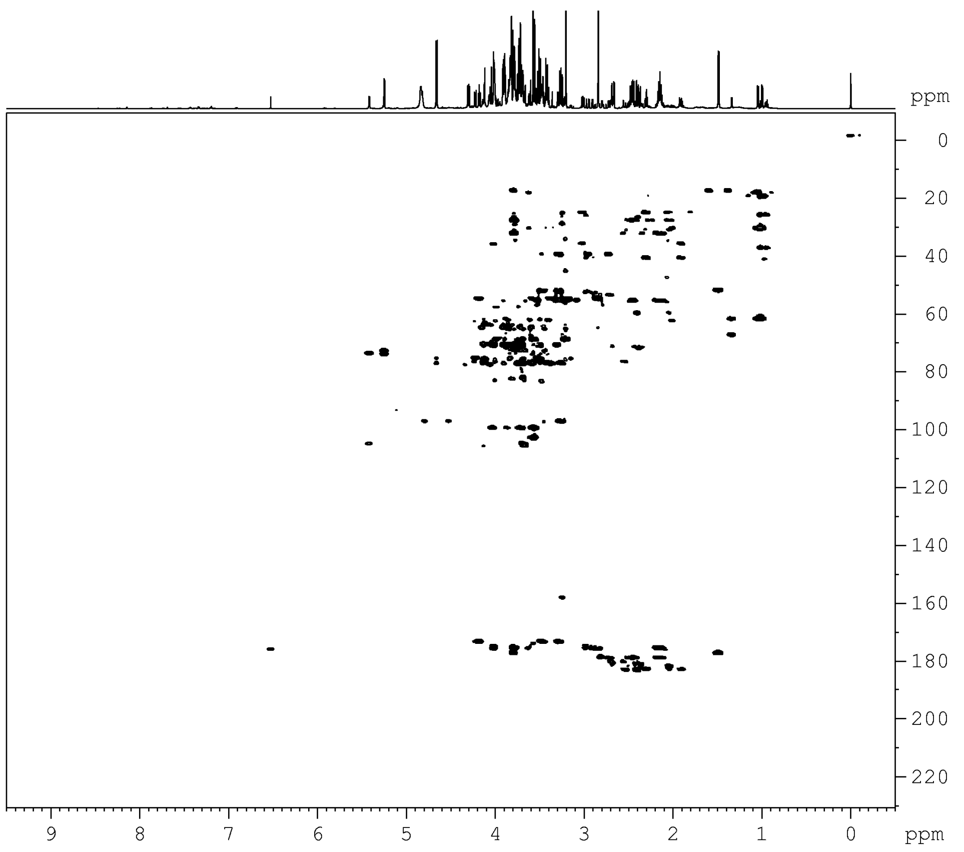

Figure S19. The ^{1}H-^{13}C HMBC NMR 2D map of cauliflower florets aqueous extract.

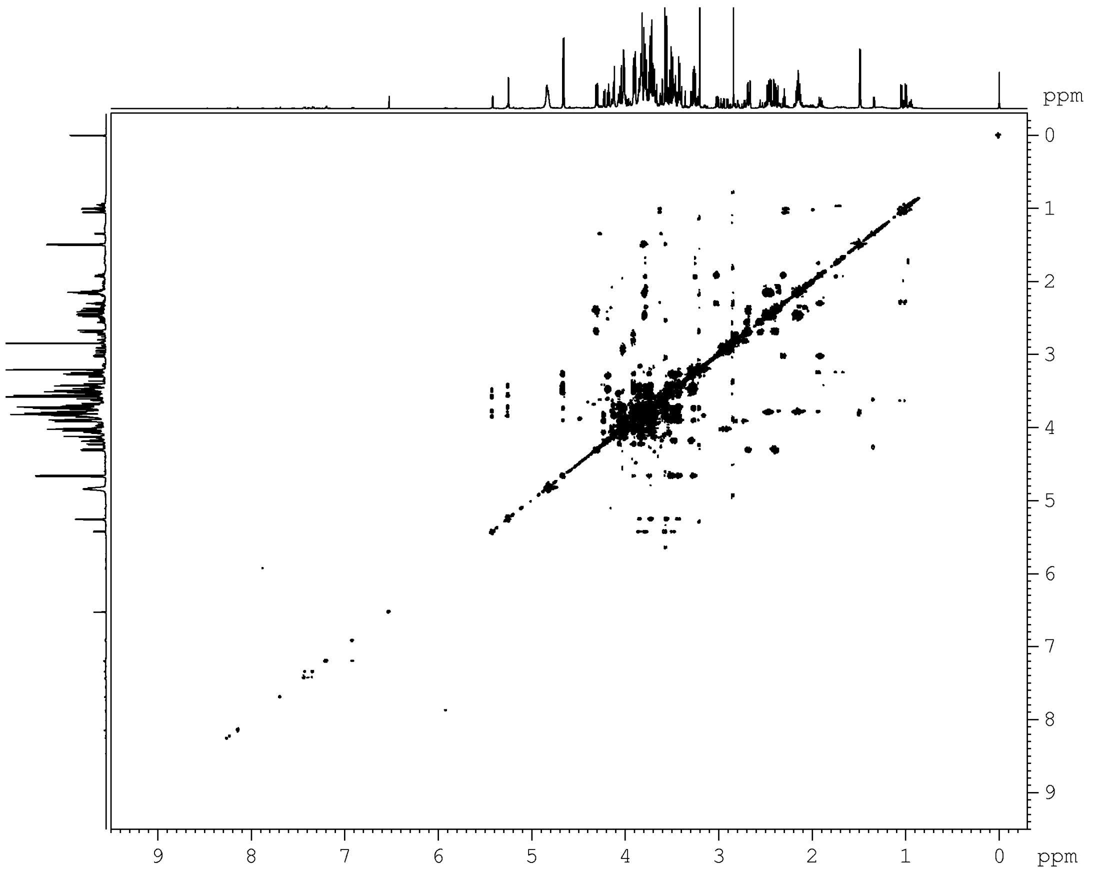

Figure S20. The ^{1}H-^{1}H TOCSY NMR 2D map of cauliflower florets aqueous extract.

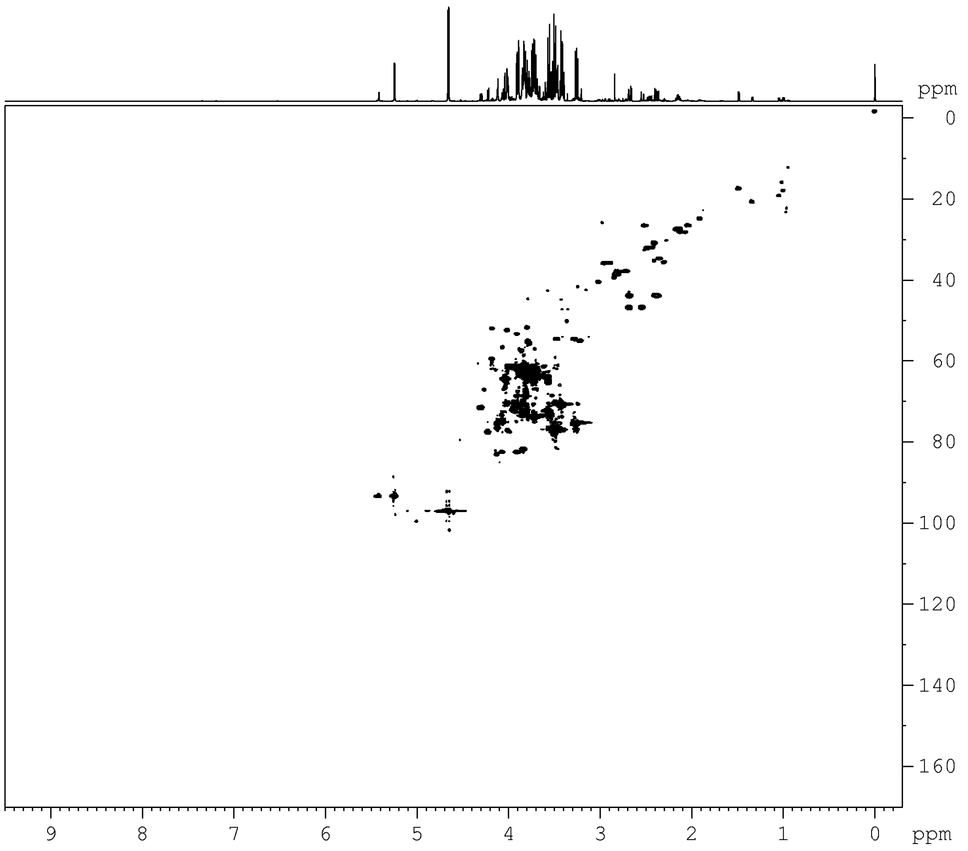

Figure S21. The ^{1}H-^{13}C HSQC NMR 2D map of cauliflower stalks aqueous extract.

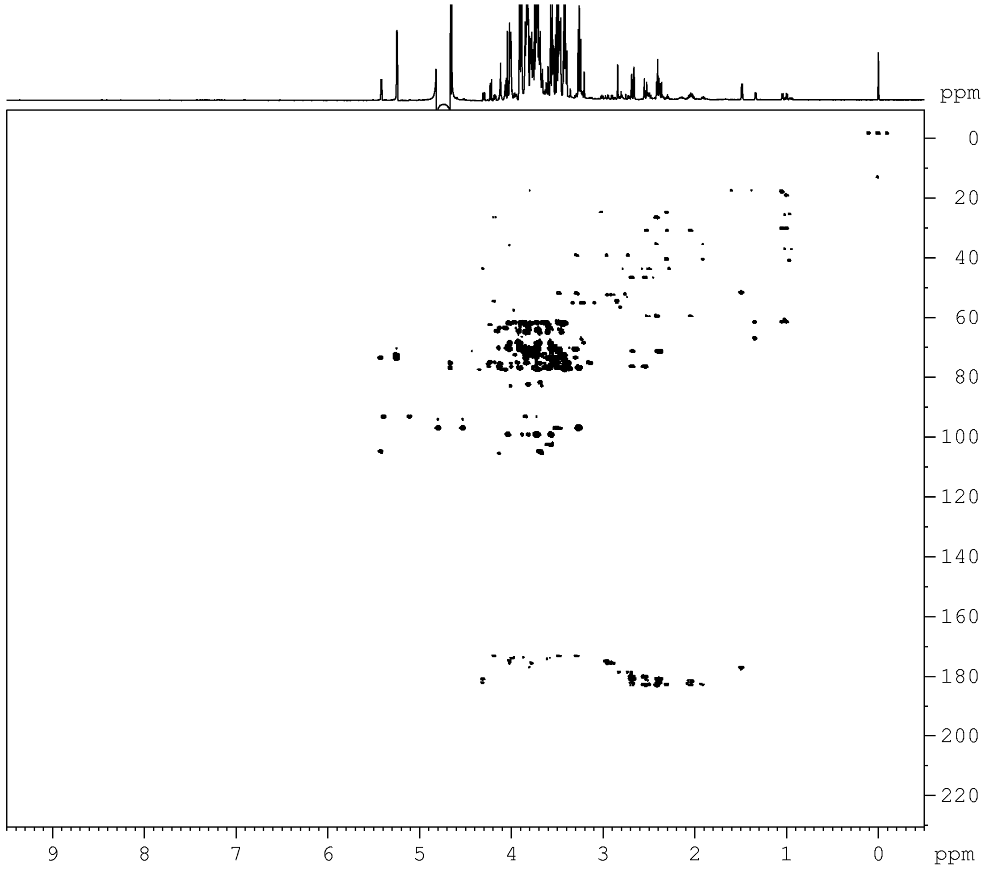

Figure S22. The ^{1}H-^{13}C HMBC NMR 2D map of cauliflower stalks aqueous extract.

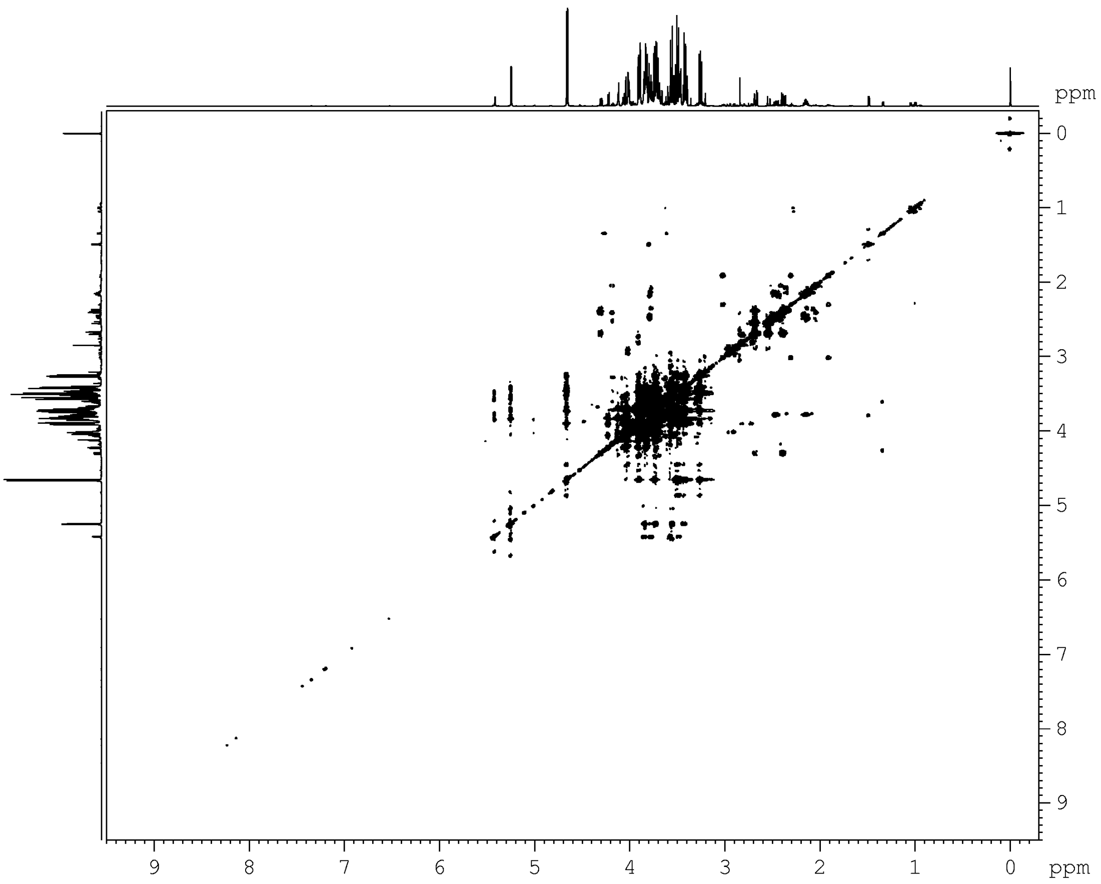

Figure S23. The ^{1}H-^{1}H TOCSY NMR 2D map of cauliflower stalks aqueous extract.

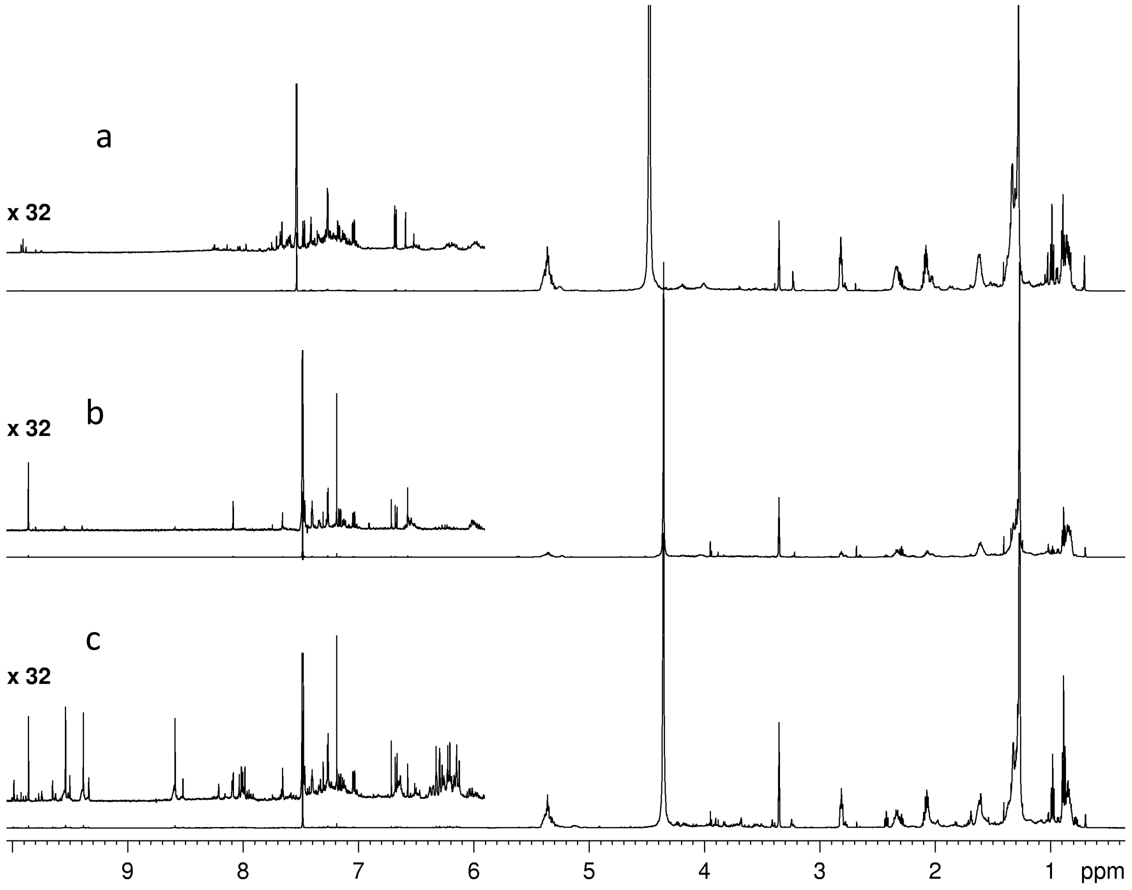

Figure S24. ^{1}H NMR spectra of cauliflower florets (a), stalks (b) and leaves (c) chloroform extracts. Solvent: $CDCl_3/CD_3OD$ 2:1 v/v.

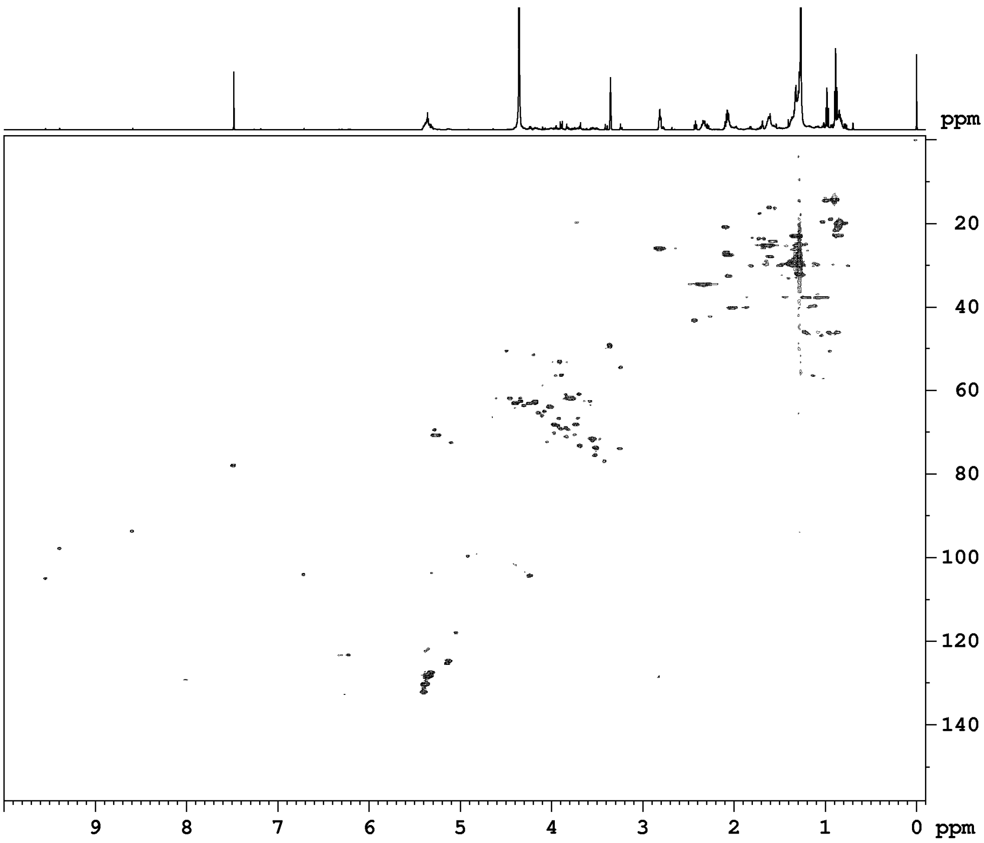

Figure S25. The ^{1}H-^{13}C HSQC NMR 2D map of cauliflower leaves chloroform extract.

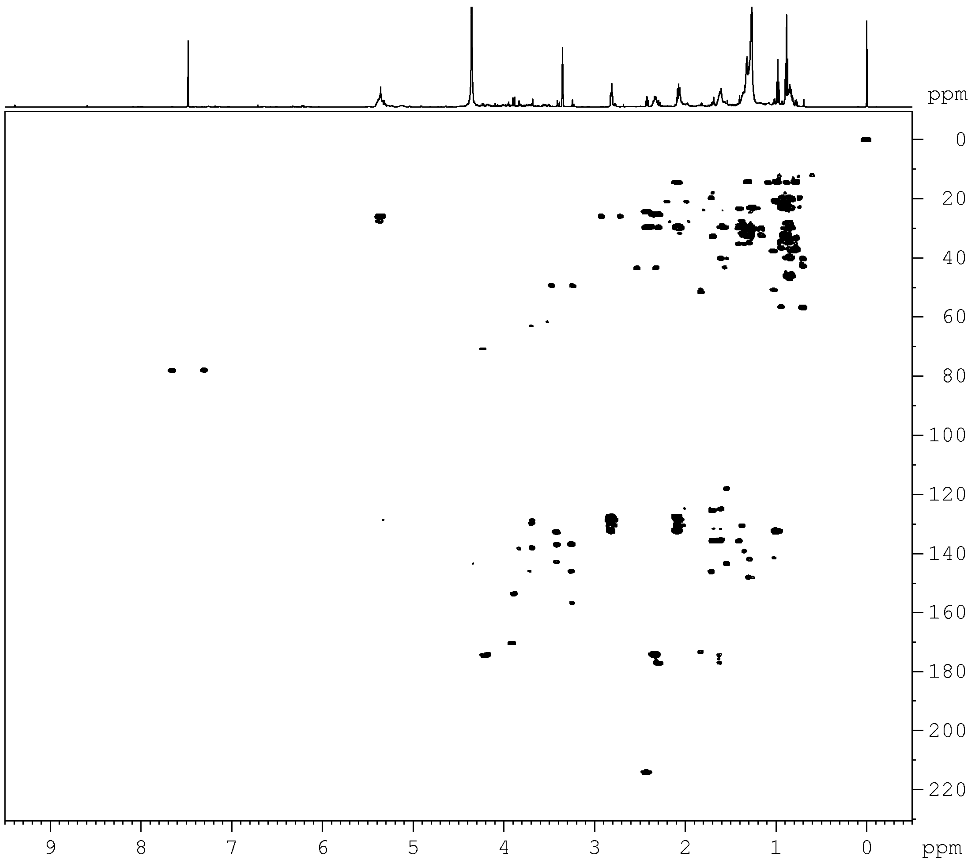

Figure S26. The ^{1}H-^{13}C HMBC NMR 2D map of cauliflower leaves chloroform extract.

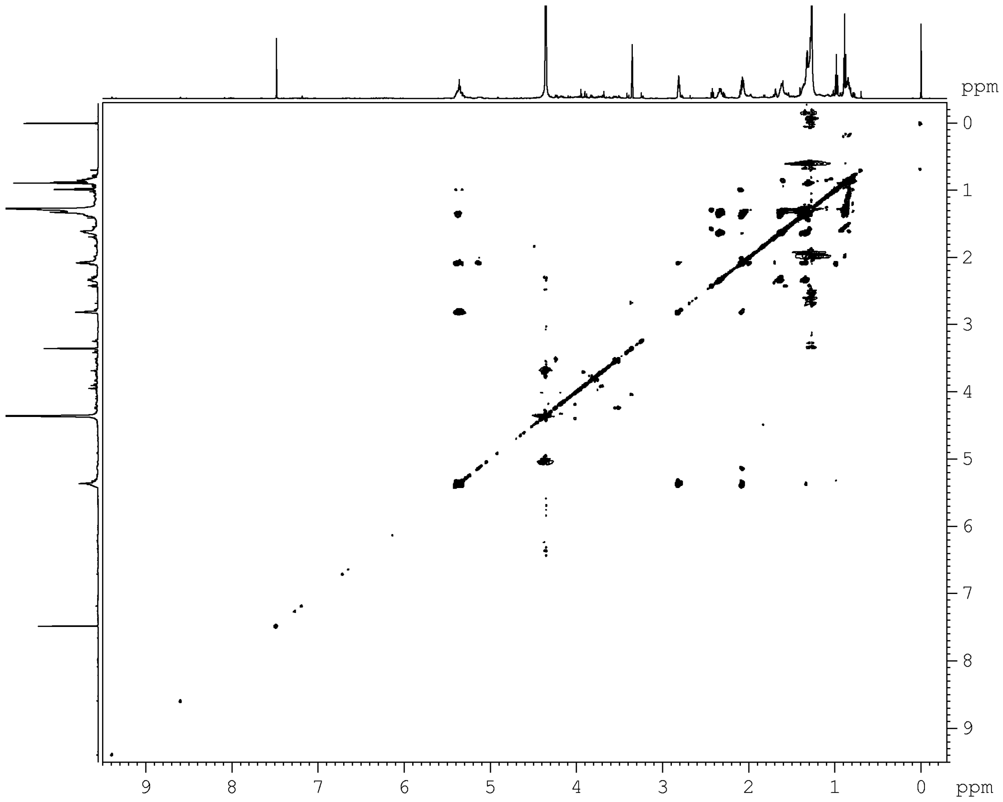

Figure S27. The ^{1}H-^{1}H TOCSY NMR 2D map of cauliflower leaves chloroform extract.

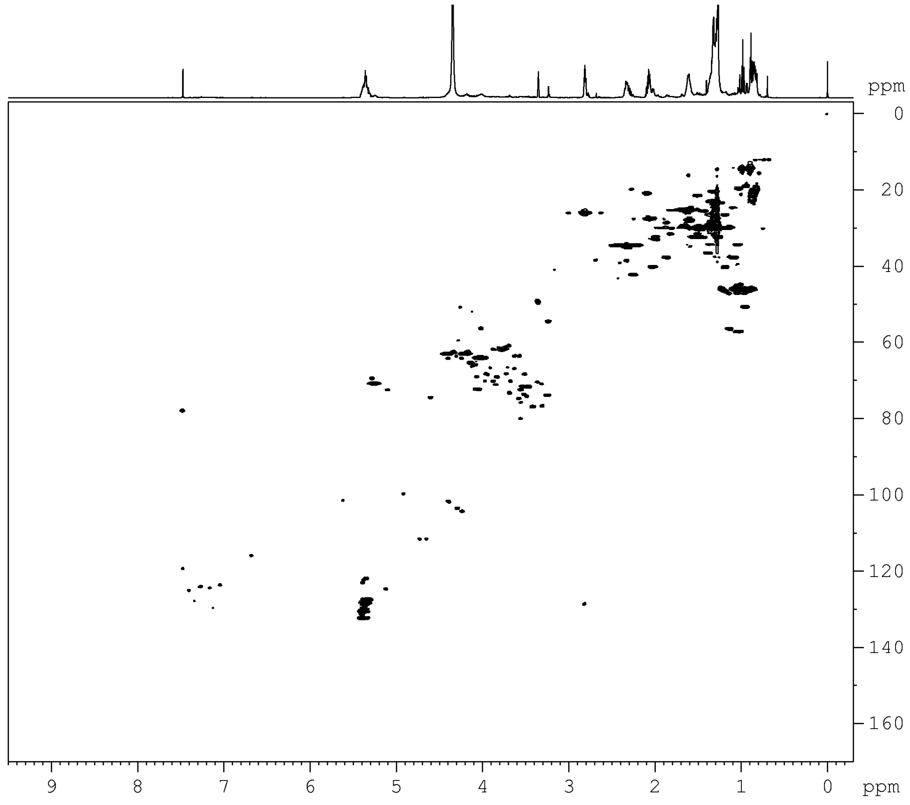

Figure S28. The ^{1}H-^{13}C HSQC NMR 2D map of cauliflower florets chloroform extract.

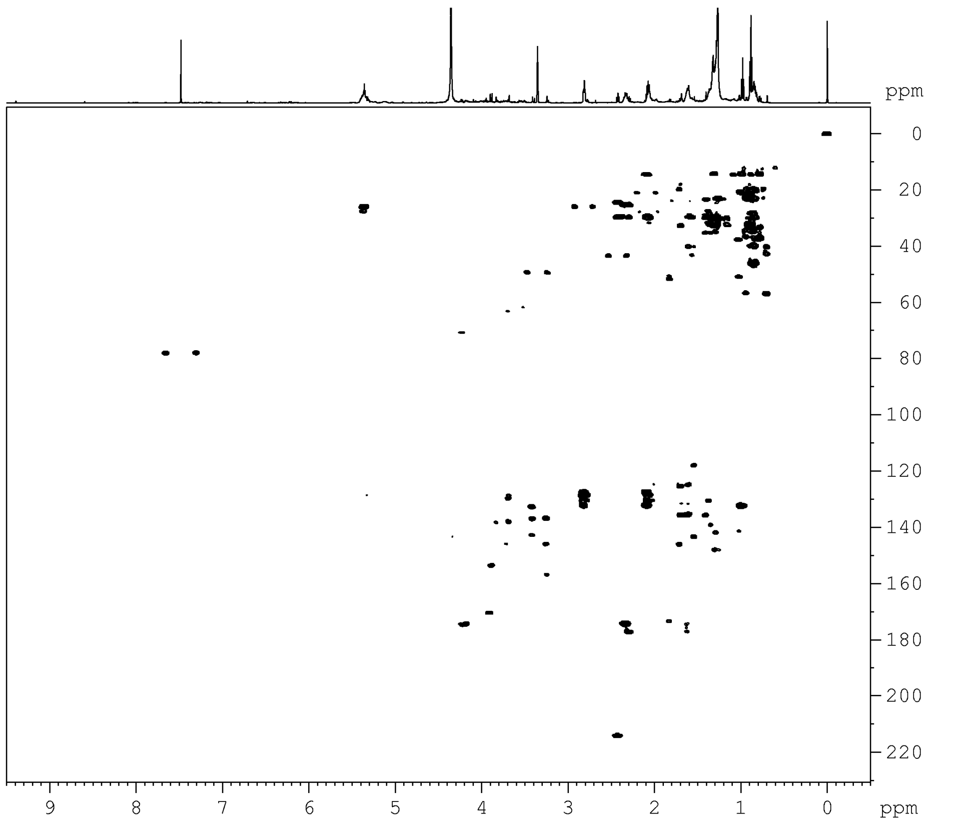

Figure S29. The ^{1}H-^{13}C HMBC NMR 2D map of cauliflower florets chloroform extract.

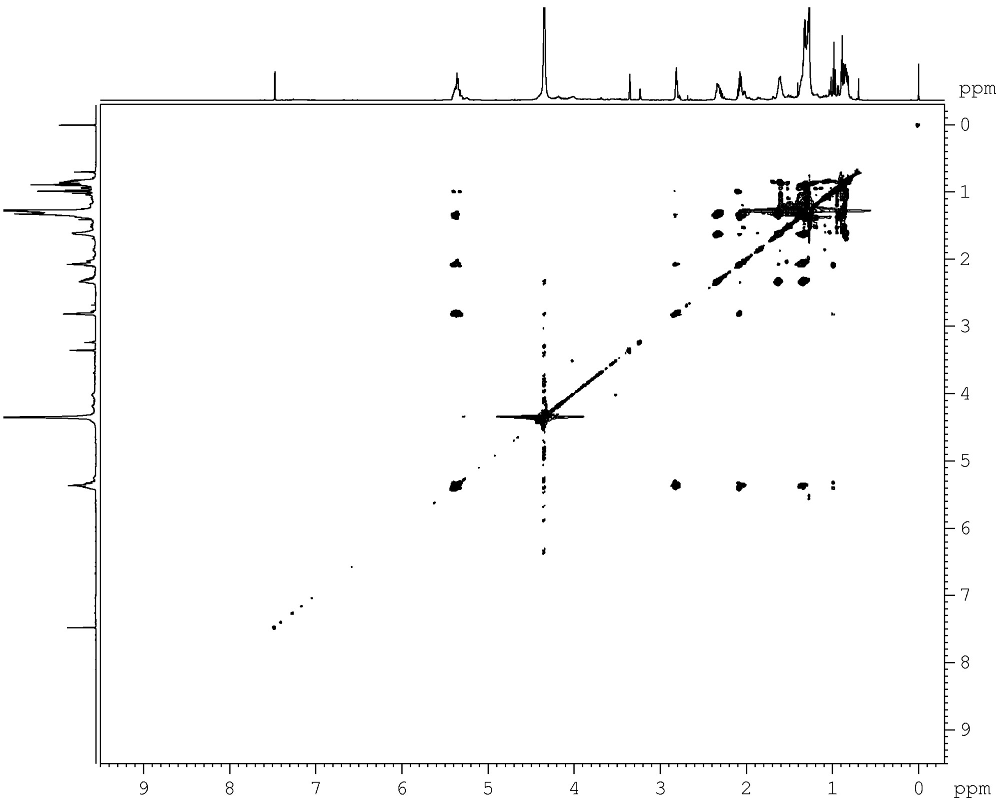

Figure S30. The ^{1}H-^{1}H TOCSY NMR 2D map of cauliflower florets chloroform extract.

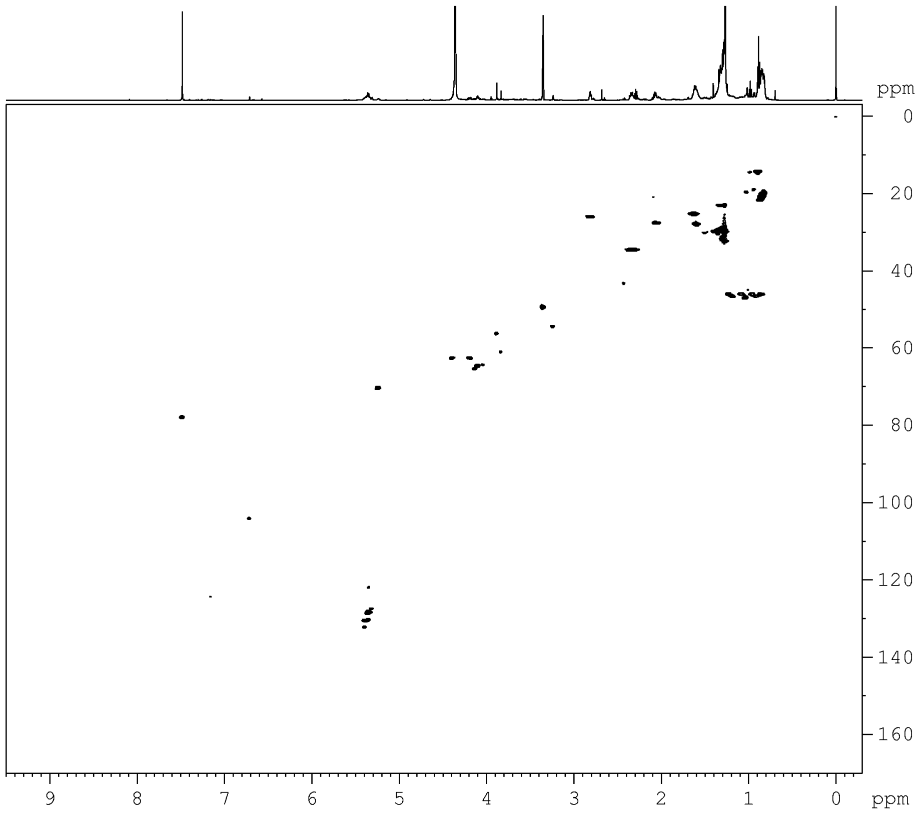

Figure S31. The ^{1}H-^{13}C HSQC NMR 2D map of cauliflower stalks chloroform extract.

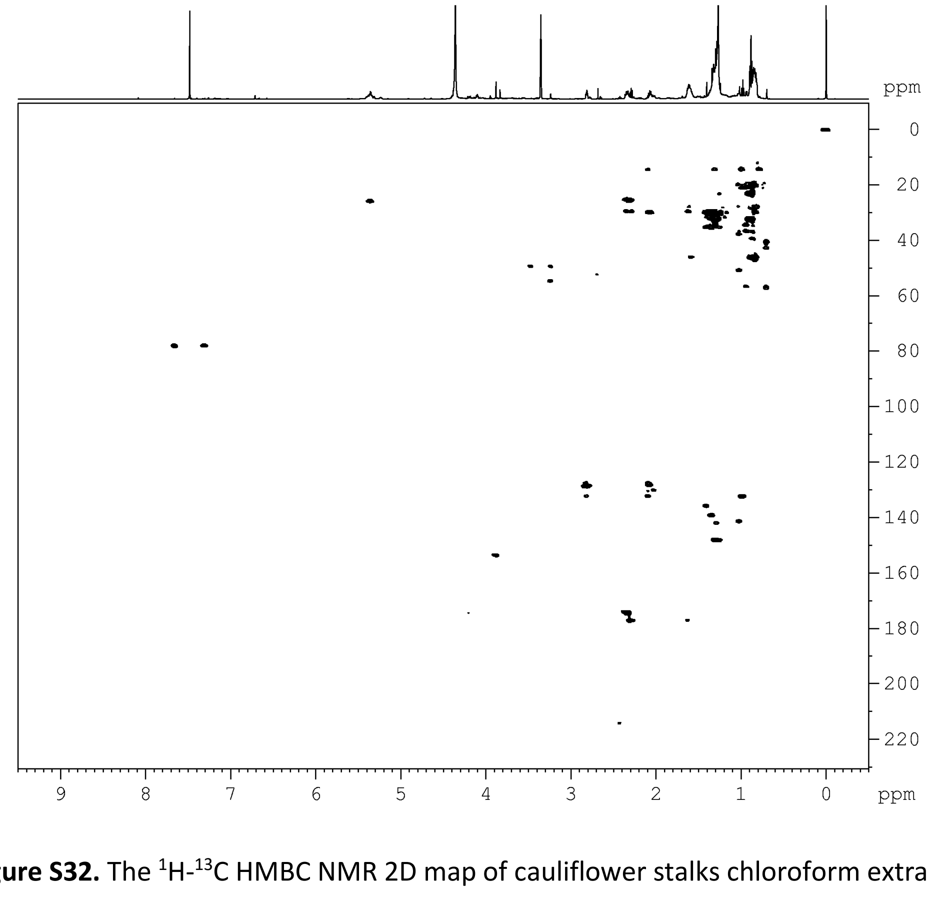

Figure S32. The ^{1}H-^{13}C HMBC NMR 2D map of cauliflower stalks chloroform extract.

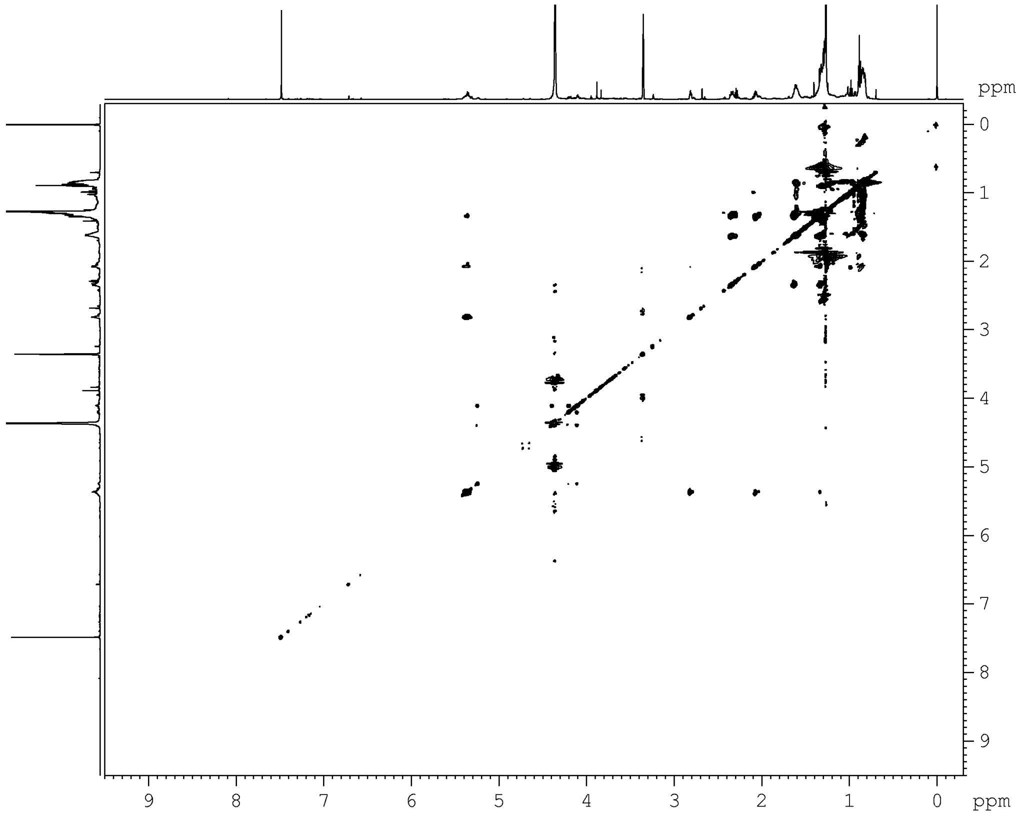

Figure S33. The ^{1}H-^{1}H TOCSY NMR 2D map of cauliflower stalks chloroform extract.
